# An eighteen-organ microphysiological system coupling a vascular network and excretion system for drug discovery

**DOI:** 10.1038/s41378-025-00933-3

**Published:** 2025-05-14

**Authors:** Jing Wang, Huixue Zhang, Yueyang Qu, Yang Yang, Shuhui Xu, Zhenni Ji, Yuxiu Wang, Xiuli Zhang, Yong Luo

**Affiliations:** 1 State Key Laboratory of Fine Chemicals, Department of Pharmaceutical Engineering, School of Chemical Engineering, Dalian University of Technology, #2, Linggong Road, Dalian, 116024 Liaoning Province China; 2 Jiangsu Key Laboratory of Neuropsychiatric Disease and College of Pharmaceutical Science, Suzhou Medical College, Soochow University, #199, Renai Road, Suzhou, 215127 Jiangsu Province China

**Keywords:** Microfluidics, Engineering

## Abstract

Physiological supporting systems, such as the vascular network and excretion system, are crucial for the effective functioning of organs. This study demonstrates that when a body-on-a-chip microdevice is coupled with miniaturized physiological support systems, it can create a multi-organ microphysiological system capable of more accurately mimicking the physiological complexity of a body, thereby offering potential for preclinical drug testing. To exemplify this concept, we have developed a model system comprising 18 types of microtissues interconnected by a vascular network that replicates the in vivo blood distribution among the organs. Furthermore, this system includes an excretory system with a micro-stirrer that ensures elimination efficiency akin to in vivo conditions. Our findings indicate that this system can: (1) survive and function for almost two months; (2) achieve two-compartment pharmacokinetics of a drug; (3) investigate the dynamic relationship between the tissue distribution and toxicity of a drug; (4) establish the multimorbidity model and evaluate the effectiveness of polypharmacy, challenging tasks with traditional animal models; (5) reduce animal usage in drug evaluations. Notably, features from points (2) to (4) are capabilities not achievable by other in vitro models. The strategy proposed in this study can also be applied to the development of multi-organ microphysiological systems that mimic the physiological complexity of human organs or the entire body.

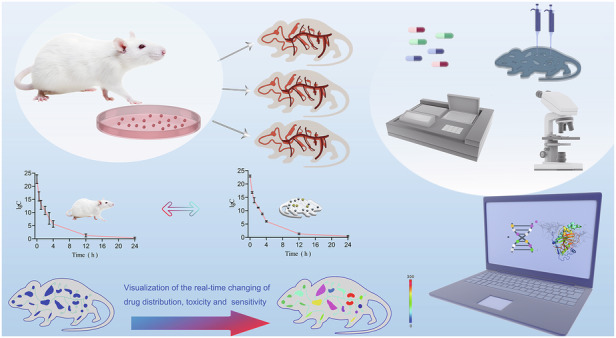

## Introduction

Drug discovery is hindered by excessively high costs^1^. To address this challenge, a range of advanced techniques, such as cryo-electron microscopy^2,3^ and aptamers^4,5^ are being utilized. Among these technologies, the microphysiological systems (MPSs)^6^ as alternative models stand out because this concept encompasses organoids^7^, organ-on-a-chip^8^, and 3D bioprinting^9^ technologies. Each technology counts for contemporary drug discovery.

Remarkably, multi-organ MPSs, characterized by their cell or cell aggregate co-culture systems, are highly valued for their ability to simulate the complex physiological landscapes of a body^10^. To date, a diverse spectrum of multi-organ MPS configurations has been documented, with dual-organ MPSs and triple-organ MPS serving as the main focus of research, such as liver-heart^11–14^, liver-skin^15,16^, liver-gut^16–26^, liver-lung^27^, liver-GI tract^28^, liver-testicle^29^, liver-pancreas^30,31^, liver-neurosphere^32^, liver-kidney^33–35^, liver-immune^36,37^, liver-trachea^38^, liver-tumor^39,40^, tumor-lymph node^41^ pancreas-muscle^42^, lung-brain^43^, tumor-heart^44,45^ and two lymph nodes^46^. Furthermore, three-organ combinations such as liver-heart-lung^47,48^, gut-liver-kidney^49^, gut-liver-immune^50^, pancreas-muscle-liver^51^, lung-liver-tumor^52^, as well as liver-heart-tumor^53^ have been reported. Additionally, four-organ combinations like liver-bone-heart-skin^54^, heart-muscle-liver-immune^55^, intestine-liver-skin-kidney^56^, lung-brain-bone-liver^57^ have been documented. Notably, more complex configurations include a seven-organ combination of liver-cardiac-lung-vascular-testis-colon-brain^58^ and gut-liver-cardiac-lung-vascular-fat-tumor^59^, and a ten-organ combination of liver-pancreas-gut-lung-heart-muscle-brain-endo-skin-kidney^60^.

These studies on multi-organ MPSs typically focus on the establishment of co-culturing systems. Researchers select specific organ types based on their objectives to conduct co-culture, enabling interactions between organ cells within this system^30,31,46^. However, due to the neglect of crucial physiological supporting systems, such as the vascular network and excretion system, as well as the limited number of organs involved, the translational relevance and physiological accuracy of these in vitro models remain limited. To address this limitation, this study introduces a strategy to couple a vascular system and excretion system to a body-on-a-chip microdevice incorporating 18 “organs”, and the applications of the resulted hybrid microdevice in the drug evaluations. Technically, we utilized rat microtissues to represent organs, opting for them over human organoids due to their straightforward acquisition through simple dissection. In contrast, acquiring such a diverse array of “organs” using human organoids presents a significantly greater challenge.

## Results

Utilizing rat microtissues to emulate organs, the resultant MPS microdevice actually serves as a simulation of a rat body. Similarly, if microtissues from other mammals were employed, the resulting MPS would represent the body of that specific mammal. Drawing inspiration from this relationship, the microdevice in this study was designed with a rat-like outline to clearly indicate its biological representation; the majority of the organ compartments were likewise outlined to correspond with their biological equivalents, and the organ compartments were roughly patterned after the anatomy of a real rat (Figure S1 in the SI).

### Fabrication of the Proposed MPS

**Fig. 1:**
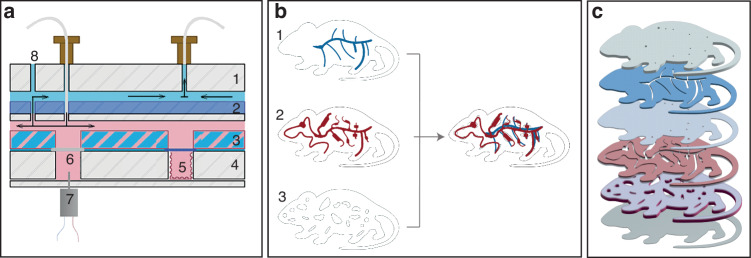
The design and fabrication of the proposed MPS. **a** The section view of the MPS. 1, cover plate; 2, “vein” plate; 3, “artery” plate; 4, “organ” plate; 5, superhydrophobic “organ” compartment covered by a porous membrane; 6, kidney-1 compartment covered by a dialysis membrane; 7, micro-stirrer; 8, ventilation port; The light blue between the “artery” plate and “organ” plate represents the hydrophobic PTFE layer. The light red between “vein” and “artery” plates indicates arterial “blood”. The light blue between cover plate and “vein” plate represents venous “blood”. The arrows indicate the flow direction inside the MPS; **b** Illustration of the top “vein” layer (1), middle “artery” layer (2) and bottom “organ” layer (3) of the MPS, and their assembly; **c** The exploded view of the MPS(The chip design details are shown in Figure S8)

This MPS was constructed through lamination (Fig. 1a), comprising “vein,” “artery,” and “organ” layers (Fig. 1b), each consisting of multiple PMMA sublayers (Fig. 1c) created by laser ablation^61^. Specifically, the “organ” layer featured 20 distinct compartments (Table S1 in the SI), of which the internal surfaces were coated with a superhydrophobic material (Figure S2-S5 in the SI). These compartments were tailored to accommodate up to 18 kinds of microtissues and waste elimination (Video-1 and Figure S6~S7 in the SI).

The assembly of this MPS began with the positioning of the “artery” layer on top of the “organ” layer, followed by the attachment of the “vein” layer onto the “artery” layer (Fig. 1b). The microchannel network in the “artery” layer and the compartments in the “organ” layer were careful aligned to ensure efficient nutrient and oxygen supply to the microtissues within the compartments. To prevent microtissues from potentially escaping upwards through the “artery,” a porous nylon membrane with a pore size of 60 μm was placed between each compartment and the “artery” (Fig. 1a). Subsequently, the microchannel network in the “vein” layer was vertically interconnected with the microchannel network in the “artery” layer at the microchannel ends.

In this study, thin layers of hydrophobic PTFE were inserted between “organ” and “artery” layers (Fig. 1c and Figure S8) to replace the double-sided adhesive tape traditionally used to bond PMMA sublayers^62^. PTFE has superior self-cleaning properties and hydrophobic characteristics which prevent any aqueous solution from leaking from the microchannels. Additionally, this PTFE-based bonding method offers improved durability and makes the microdevice detachable and reusable, as well as facilitates retrieval of the microtissues and their supernatants.

There was a micro-stirrer (DC motor 610 with variable input power) presented in the kidney-1 compartment (Fig. 1A). Videos 2-4 in the SI showed the varying grades of mixing in the compartment. It was demonstrated that this micro-stirring could increase the rate of mass transfer between the kidney-1 compartment and the “artery” (Figure S9~S11 in the SI). A specialized cartridge was created specifically for culturing this MPS (Video-5 in the SI).

### Characterization of the Proposed MPS

#### The “Blood” Circulation

**Fig. 2:**
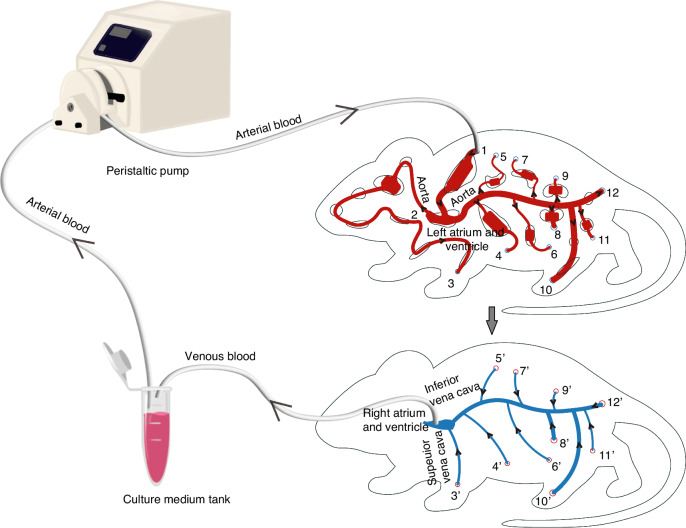
Illustration of the “blood” circulation system of the proposed MPS. Flow direction: peristaltic pump——1——2——(3, 4, 5, 6, 7, 8, 9, 10, 11, 12) ——(3’, 4’, 5’, 6’, 7’, 8’, 9,’ 10’, 11’, 12’) ——culture medium tank——peristaltic pump

As depicted in Fig. 2, the “arterial blood” flow is driven by a peristaltic pump from the culture medium tank to the “lung,” subsequently proceeding to the “left atrium and ventricle” and then to the “aorta.” After perfusing all compartments, the “arterial blood” transforms into “venous blood,” which then moves vertically into the “vein” layer. It converges at the “right atrium and ventricle” via the “superior vena cava” and the “inferior vena cava,” ultimately returning to the culture medium tank for re-oxygenation, thereby completing one cycle of “blood” circulation within the system. The merits of this “blood” circulation design stem from its close resemblance to the in vivo blood circulation pattern. Additionally, our preliminary findings have indicated that, in a body-on-a-chip system, the arrangement of these organs can potentially impact their functionality. Therefore, it is advisable not to simply adopt a straightforward serial manner in multi-organ chip systems.

The distribution of “blood” flow across the “organs” is primarily governed by the numerical design of the vascular network (Eq. 1-74 in the SI). Theoretically, by adjusting the dimensions of the microchannel network, we can achieve a “blood” flow distribution across the “organs” that closely mimics in vivo conditions. Finally, we found that, with the dimensions outlined in Equations 75-146. Both in theoretical calculations and experimental measurements, the “blood” flow distribution across various “organs” is consistent with the results obtained in vivo. (Video-6 & Figure S12~14 in the SI, and Table 1).

**Table 1:** “Blood” flow proportion at each “organ”

Organ	Calculated “blood” flow proportion in this MPS	Measured “blood” flow proportion in this MPS	“Blood” flow proportion in vivo^82^
Heart	100%	100%	100%
Lung	100%	100%	100%
Liver	26.79%	24.39%	25%
Brain (Eye, ear, Nose, Tongue, Trachea, adipose, muscle)	12.22%	12.98%	12%
Stomach	4.08%	5.65%	1%
Pancreas	1.96%	3.57%	1%
Kidney	2.67%	3.01%	–
Kidney-1	30.31%	29.11%	25%
Spleen	3.47%	4.55%	3%
Skin	4.71%	3.08%	5%
Marrow	4.71%	3.08%	5%
Testicle	12.98%	12.77%	–
Tumor	0.81%	0.89%	–

#### The Excretion System

**Fig. 3:**
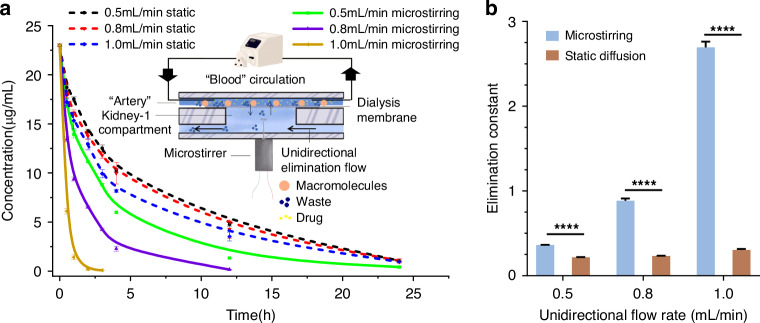
The performance of the excretion system of the proposed MPS. **a** Variation of the elimination curve of sodium fluorescein with the unidirectional flow rate in the presence and absence of microstirring (3 v). The center is the illustration of the excretion system in this MPS; **b** The elimination constants calculated from A frame. (*n* = 3, ****p < 0.0001)

Within this MPS, two kidney compartments were present. Located at the back, the kidney compartment housed kidney microtissues subject to drug tests. The kidney-1 compartment in the belly held only culture medium and was used for the elimination of body waste and drugs. The central part of Fig. 3a depicts the design of the excretion system. A dialysis membrane was sandwiched between the kidney-1 compartment and “artery”, allowing for the diffusion of small molecules from the “artery” to the kidney-1 compartment and their eventual elimination through the unidirectional culture medium flow beneath the kidney-1 compartment. The micro-stirrer in the kidney-1 compartment can enhance the efficiency of the mass transfer process. Thus, the elimination profile and rate of small molecules can be adjusted by either the unidirectional flow beneath the kidney-1 compartment (Fig. 3a) or the micro-stirring in the compartment (Fig. 3c).

#### The Vitality

**Fig. 4:**
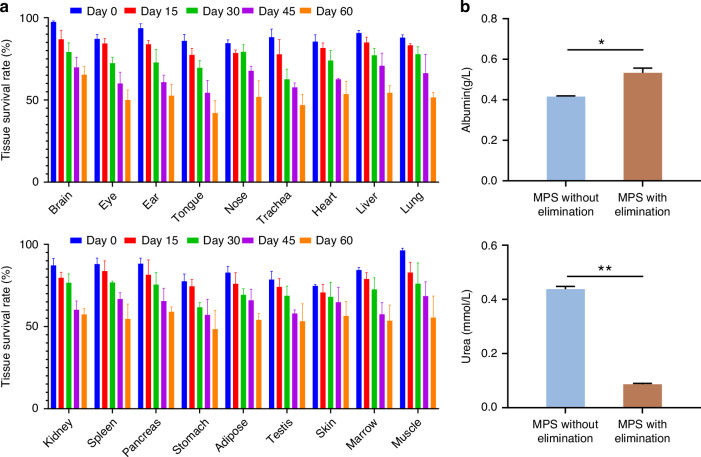
The vitality of the proposed MPS. **a** Tissue survival rate over two months; **b** The variation of albumin (top part) and urea (bottom part) levels in the MPSs with and without kidney-1 clearance. (*n* = 3, *p < 0.05, **p < 0.01)

This MPS offers several advantages compared to traditional microfluidic co-culture systems, (1) It is capable of integrating up to 18 types of microtissues, a feature not commonly found in other methods; (2) It utilizes a customized culture medium (Table S2 in the SI) and collagenase treatment (Figure S7 in the SI) for freshly-cut microtissues, thereby increasing their viability; (3) Additionally, it employs micro-mixing techniques to enhance mass transfer between the kidney-1 compartment and “artery”, while exploiting unidirectional flow to eliminate detrimental metabolites. All of these measures greatly improve the microtissues’ access to nutrition and oxygen and ensure the elimination of wastes, thereby ensuring their long-term viability. In Fig. 4a, our hypothesis is confirmed, demonstrating that the lifespan of this MPS can reach up to 2 months, even though the viability of microtissues is left by approximately 50% at the end. Additionally, Fig. 4b illustrates that the culture medium in this MPS exhibits higher levels of albumin and lower levels of urea, further substantiating the higher vitality of this MPS compared to traditional co-culture systems. It is noteworthy that when primary cells are cultured in vitro, there is a risk of a small number of cell deaths triggering rapid and widespread cell death, somewhat akin to a “domino effect” chain reaction. However, this phenomenon did not occur in this MPS. This phenomenon can be attributed to this MPS’s excretion system, being capable of elimination of small-molecular wastes (e.g. urea) while maintaining cell-cell interaction factors (e.g. albumin) within the system.

### Applications of the Proposed MPS in the Drug Evaluation

This paper used carboplatin as model drug to show the capability of this MPS in the field of drug evaluation.

#### Evaluation of the Pharmacodynamics

**Fig. 5:**
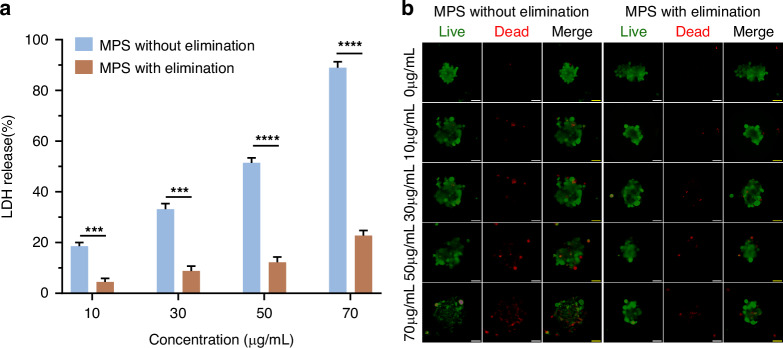
The efficacy of carboplatin on the tumor in the proposed MPS with and without kidney-1 clearance. **a** LDH activity measurement (*n* = 3, ***p < 0.001, ****p < 0.0001); **b** Live/dead staining (scale bar:100 µm)

Firstly, we followed a method described in a previous study^63^ to prepare the tumor, and then transformed into microtissues, which were loaded into this MPS. Subsequently, carboplatin was injected into this MPS, allowing it to circulate within the culture medium for a duration of 3 days. The efficacy of carboplatin was then evaluated. As depicted in Fig. 5a and b, the dose-responsive characteristics of carboplatin’s effectiveness were found to be consistent with the prior report^64^. Notably, it is crucial to highlight that the reduction in viability observed within this MPS was less pronounced when compared to normal co-culturing conditions. This observation underscores the significance of the excretion system present within this MPS, which contributes to properly assess the exposure of drugs to facilitate the estimation of clinical doses.

#### Evaluation of the Pharmacokinetics

The multi-organ microphysiological system serves as a suitable tool for conducting in vitro pharmacokinetic research^59^. In vitro pharmacokinetic research^65–67^ serve as an important technical support for early ADME research, enabling the elimination of candidate drugs with potentially unfavorable pharmacokinetic properties at the early stages of new drug development. This helps to improve the success rate of new drug research and development. A previous study utilized three-organ chips and computer simulations to obtain the pharmacokinetic parameters of cisplatin^49^. Because only a few “organs” were used and the type and quantity of “organs” used need to be adjusted according to the drug candidates, the wide applicability of this method was compromised.

**Fig. 6:**
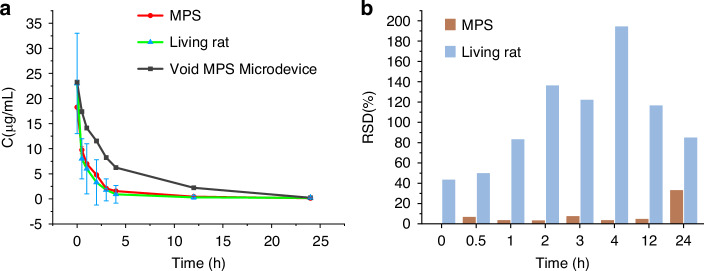
Pharmacokinetics of carboplatin in the proposed MPS loaded with and without microtissues. **a** The drug-time curves of carboplatin on varied occasions. In the MPS experiments, *n* = 4. In rat experiments, *n* = 4. In void MPS microdevice experiment, *n* = 1; **b** the RSD of data points in the drug-time curves of carboplatin on varied occasions

In our study, we employed the 18-organ MPS to measure the drug-time curve of carboplatin. We compared this curve with the one obtained from rats^68^, and Fig. 6a demonstrates a strong agreement between this MPS and rat curves, indicating the equivalence of the MPSs as a model to rats. Notably, this MPS curve exhibited distinct two-exponential decays, suggesting two-compartment characteristics. Through numerical simulations, we determined that this MPS curve can be described by the equation $C=6.187{e}^{-4.794t}+12.074{e}^{-0.517t}$. From the above equation, some pharmacokinetic parameters of carboplatin can be calculated, for example, the half-life (t_1/2β_) of carboplatin obtained from 18-organ MPS is 1.34 h, within the scope of the literature report^69,70^. This marks the first instance where an in vitro model successfully replicates two-compartment drug-time curve.

Two-compartment pharmacokinetics is the dominant model for most drugs within the body^71^. Our MPS technology replicates this model due to two main reasons: first, this MPS “blood” flow distribution closely mimics that of a real rat, and second, it incorporates 18 microtissues. Figure 6a illustrates that when this MPS does not contain microtissues, the drug concentration-time curve follows a one-exponential decay consistent with a one-compartment model. However, when microtissues are included, the drug-time curve undergoes deformation, transforming into a two-compartment model. This fact indicates the sufficient interaction between drugs and 18 “organs” in this system.

One significant reason for the huge consumption of laboratory animals is their inherent variability. For instance, not all laboratory rats can develop fatty liver even under a high-fat diet^72^. Individual differences among animals also manifest in pharmacokinetics, where high variability in drug concentration at each time point leads to a large relative standard deviation (RSD) and extensive animal usage. Figure 6b demonstrates that, on average, the RSD in laboratory rat experiments is more than 10 times higher than that in the MPSs (*n* = 4), implying the potential of reduction in rat consumption if using this MPS in replacement of living rat.

#### Evaluation of the Dynamic Relationship between the Tissue Distribution and Toxicity

The distribution of drugs among “organs” directly affects their tissue toxicity in clinical settings. While the relationship between distributions and toxicities at a specific time point can be studied by euthanizing animals and analysing their organs, it is challenging to measure their dynamic relationship. This is because one animal cannot be euthanized more than once, which limits our ability to gain a more in-depth understanding of the nature of drugs. However, the introduction of the MPSs can help address this issue. The MPSs possess nearly identical characteristics, enabling parallel experiments and providing valuable insights into the dynamic relationship between distribution and toxicities.

**Fig. 7:**
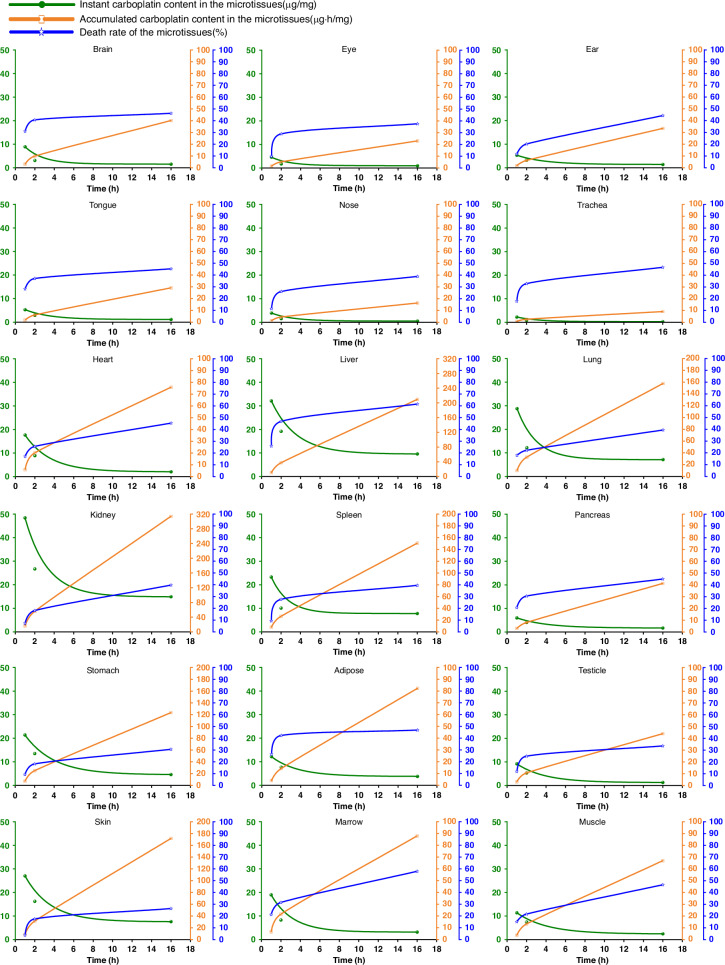
Relationship between instant/accumulated tissue distribution and tissue toxicity of the carboplatin. Green curve: instant carboplatin concentration (μg/mg tissue); orange curve: accumulated carboplatin exposure (μg h/mg tissue); blue curve: microtissue death rate (%)

We conducted an experiment involving the dissection of two rats and mixing their microtissues to obtain the six identical MPSs. Three MPSs were used to assess the carboplatin distribution at three specific time points (shown as green lines in Fig. 7). Another three MPSs were used to measure the viability of various tissues at the same time points (represented by blue lines in Fig. 7). By integrating the green lines (indicated as yellow lines in Fig. 7), we were able to determine the accumulated concentration of carboplatin in these tissues.

**Fig. 8:**
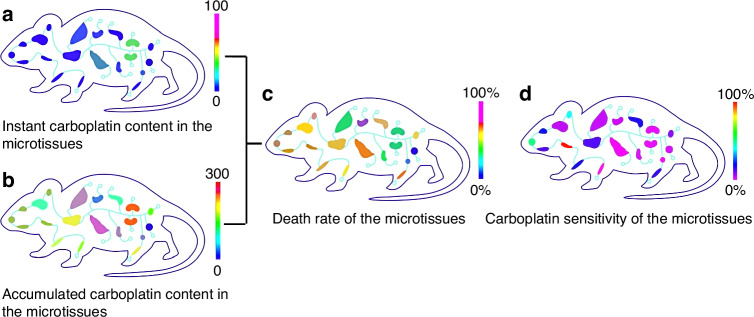
Heatmaps of instant, accumulated carboplatin concentrations, carboplatin tissue toxicity, and carboplatin sensitivity of the microtissues. **a** Instant carboplatin content in the microtissues. **b** Accumulated carboplatin content in the microtissues. **c** Death rate of the microtissues; **d** Carboplatin sensitivity of the microtissues

Through the curves in Fig. 7, we can observe differences among 18 “organs” in terms of instant drug content, accumulated drug content, and death rate. The instant drug content curves of the 18 “organs” exhibit variations, even though their concentrations at the endpoint of 16 hours are similar. The peak concentration of the drug in an organ, Cmax, is also related to whether the drug exhibits toxicity. This result indicates the importance of assessing drug tissue distribution at different time points. These parameters and their variation dynamics can be visualized by heat maps (Fig. 8a, b, c and Video-7 in the SI). By comparing the ratio of accumulated drug content and death rate slopes, we can identify drug sensitivity of each microtissues (Fig. 8d). This approach not only directly illustrates the differences in drug retention among various “organs” but also enhances our understanding of the interplay among pharmacokinetics, pharmacodynamics, and toxicity. It is noteworthy that we achieved these results using only six MPSs, equivalent to the use of two real laboratory rats. In contrast, traditional methods typically require the sacrifice of 20 to 50 rats. This highlights again the efficiency and effectiveness of the MPSs in reducing the animal use.

Upon careful analysis of Fig. 7, we observed a favourable distribution of carboplatin in the kidneys, liver, and spleen, which is consistent with existing literature^73^. In terms of toxicity, carboplatin demonstrated elevated toxicity in the marrow and liver, with a particular emphasis on increased toxicity against the marrow. Additionally, we noticed significant “organ” toxicity in the trachea from both Figs. 7 and 8d, even at relatively low accumulation levels. This indicates a tendency for carboplatin to induce cellular toxicity in this organ. Considering that the ratio of tissue blood flow in the human trachea is considerably higher compared to that in rodents, it is plausible that carboplatin exhibits apparent toxicity in the respiratory tract. This finding aligns with occasional observations of respiratory toxicities in the clinical use of carboplatin.

It is worth noting here that the MPSs have unique advantages for measuring the drug tissue distribution. Tissue distribution is influenced by various factors, including cellular characteristics within organs, organ weight, and blood flow. For example, in rodents, the brain tissue comprises approximately 1-2% of their body weight, while in primates, it is approximately 2-3%, which is closer to the proportion in humans. However, continuously monitoring tissue distribution depends on lots of mammals for averaging due to the considerable individual variability among natural mammals. Therefore, continuous monitoring of drug tissue distribution is typically conducted using rodents and rarely with larger mammals like monkeys. Nevertheless, with the inception of this MPS, there is potential for continuous monitoring of drug distribution in large mammals, such as monkeys, which can enhance our understanding of medications.

#### Evaluation of the Effectiveness of Polypharmacy against Multimorbidity

Multimorbidity characterized by the presence of multiple chronic conditions, is a prominent global health issue that substantially impacts an individual’s health and overall well-being^74^. However, the lack of an established animal model for studying multimorbidity has been a significant challenge. One factor to consider is that multimorbidity predominantly affects older individuals, necessitating the use of aged animals to create an appropriate model. Additionally, creating a multimorbidity model requires the introduction of multiple pathogens, which can be particularly challenging for older animals to withstand.

Nevertheless, the establishment of an animal multimorbidity model becomes more achievable with the use of this MPS. For instance, we combined brain microtissues from elderly rats with Parkinson’s disease, liver microtissues from elderly rats with carbon tetrachloride-induced liver injury, and microtissues from various organs such as the heart, lungs, kidneys, muscles, pancreas, adipose tissue, and spleen from normal elderly rats. This allowed us to successfully create an old rat model presenting both Parkinson’s disease and liver injury (The feasibility verification of this in vitro multi-disease model is shown in Figure S15).

In real life, many elderly individuals may take natural products. The targets of natural products are unknown and are often reported to have apparent therapeutic effects in different animal disease models. For example, piperine has a certain effect on Parkinson’s disease^75^, dihydroquercetin can protect the liver^76^, and catalpol can prevent aging^77^. Studies have shown that dihydroquercetin and catalpol also have neuroprotective effects^78,79^, and piperine and catalpol also have been reported to have liver protective effects^80,81^. Due to different modeling and administration methods, it is difficult to quantitatively compare their comprehensive effects based on previous research results. We utilized this multimorbidity model to quantitatively assess the effects of three natural products on three aspects of comprehensive therapeutic effects.

**Fig. 9:**
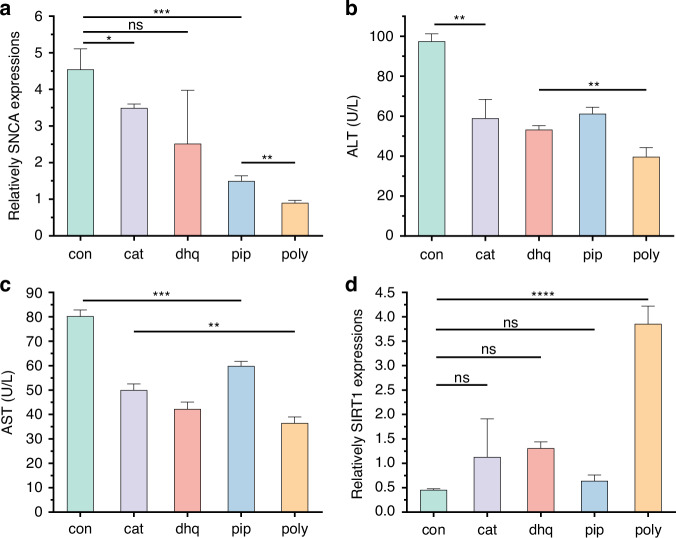
The evaluation of polypharmacy based on the proposed MPS multimorbidity model. cat, dhq, pip and poly indicated catalpol, dihydroquercetin, piperine and (catalpol+dihydroquercetin+piperine), respectively. **a** Effect of various drugs on SNCA expressions in “brain” of this MPS; **b** Effect of various drugs on ALT expressions in “liver” of this MPS; **c** Effect of various drugs on AST expressions in “liver” of this MPS; **c** Effect of various drugs on SIRT1 expressions in “kidney” of this MPS. (*n* = 3, ns indicates *p* > 0.05, *p < 0.05, **p < 0.01, ***p < 0.001, ****p < 0.0001)

Figure 9a showed that piperine displayed the lowest expression of SNCA and had significant differences compared to the control group and the other two compounds, indicating that piperine offers the most effective neuroprotective effects. Figure 9b and c demonstrated that all three compounds effectively reduced ALT and AST, indicating liver protective effects, with minimal differences between them. Similarly, in terms of anti-aging effects, the three compounds did not show a significantly greater promotion of the marker SIRT1 compared to the control group. Therefore, among the three natural products, choosing piperine could provide the maximum benefit from both brain and liver perspectives. We also compared the efficacy of the three drugs when used in combination and found that the combination therapy resulted in better outcomes in brain protection, liver protection, and anti-aging effects compared to the best single drug therapy. Particularly, there was a synergistic effect in promoting the expression of the anti-aging marker SIRT1 (Fig. 9d). Based on these results, for elderly patients with Parkinson’s disease and liver disease, the three-drug combination therapy may offer a more effective treatment approach.

## Discussion

The multi-organ MPS developed in this study can fully meet the co-culture requirements of 18 kinds of microtissues. The system can effectively simulate the complex physiological structure and function of animals through the multi-microtissue co-culture mode. Based on this, we have successfully built a multi-disease model in vitro, carried out drug toxicity studies from multiple dimensions, and efficiently screened potential drugs, providing key technical support for life science and pharmaceutical research and development.

In exploring the MPS of multi-organ interaction, the problem of cell source has become one of the key factors restricting the development of organ-on-a-chip. The use of existing cell lines to construct an organ-on-a-chip will face the challenge of diverse cell lines, which may affect the reliability of experimental results. Tissue culture in vitro is considered a transitional method between animal experiments and cell culture. It can retain all kinds of cells and tissue-specific extracellular matrix components in the tissue, so as to reflect the situation of the overall tissue to a certain extent. In this study, considering that this is a conceptual study of platform construction, it is feasible and reasonable to choose microtissues for simulation. As a simplified organ model, microtissues can reduce the complexity and cost of experiments while retaining some key characteristics of tissues. Through the integration and cultivation of multiple microtissues, we can initially explore the mechanism of multi-tissue interaction.

In common in vitro model studies, it is challenging to accurately analyze and visualize the relationship between drug distribution and drug toxicity. In this study, this multi-organ MPS can successfully achieve intuitive visualization of the relationship between drug toxicity and distribution, such as in video 7 in Supplementary Information. This visualization provides a new insight into the mechanism of action of drugs, allowing researchers to clearly observe the distribution dynamics of drugs in different tissues and how toxicity changes over time. At the same time, we explore further at the theoretical level to develop a theory describing this relationship (see SI section 1.5). The proportion of the concentration of the accumulated drug in the tissue to the death rate of the tissue can be expressed by the following equation:

1 $$Q=\frac{\rho V{\int }_{0}^{t}{V}_{c}{d}_{t}}{(1-q){\int }_{0}^{t}\left\{{\oiint}_{0}^{S}{\int }_{0}^{t}\left[\int -\frac{\partial C(x,t,S)}{\partial t}{dx}\right]{dt}\cdot {dS}\right\}{dt}}\,[x=0]$$

in which $C(x,t,S)$ is determined by,

2 $$\frac{\partial C(x,t,S)}{\partial t}=D\frac{{\partial }^{2}C(x,t,S)}{\partial {x}^{2}}$$

3 $$D={D}_{{Dif}}+{D}_{{Act}}$$

where $\rho$ is the density, $V$ is the volume of a single cell, ${V}_{c}$ is the cell death rate, and $t$ is the time, $q$ is the proportion of the extracellular matrix in the organ, $S$ is the “blood” vessel wall in the organ, $x$ is the diffusion distance, $D$ is the apparent diffusion coefficient including passive diffusion term$,{D}_{{Dif}}$ and active transportation term, ${D}_{{Act}}$.

It is noteworthy that this MPS is lack of immune system, which means this MPS is especially suitable for simulation of nude rat that has wide applications in biomedical area. Thymus-free nude mice, which are often used to study non-immunological experiments or to avoid interference from immune rejection, are deficient in T cell immune function due to the congenital absence of the thymus gland. Our MPS can well simulate their physiological environment in this respect, providing an effective platform for related research. When the application of this MPS involves an immune response, we take the approach of primary immune cells extracted from rat blood and add these cells to the circulatory system of the system. These immune cells can realize circulation in the simulated “blood” environment, so as to more realistically simulate the circulation state of immune cells in the biological body. This information is included in section 1.6 of the Supplementary Information (Figure S16-S19). In this way, it can compensate for the lack of immune function of the system to a certain extent, so that it can be used to study drug reaction processes involving immune cells. Furthermore, a limitation of the current MPS lies in the absence of an integrated lymphatic component. While this configuration suffices for the present investigation, future studies aiming to interrogate more sophisticated aspects of immune system regulation would require the incorporation of a dedicated lymphatic subsystem—a technological challenge that remains unaddressed in current multi-organ MPS platforms.

This MPS can be customized to reflect specific age groups. Assuming an original natural animal is 2 years old, the derived MPSs can accurately mirror the physiological attributes of a natural mammals of the same age. As a result, the MPSs can effectively simulate various stages of mammal’s life, including neonatal, infant, child, adolescent, adult, and elderly stages. Furthermore, the MPSs open up new possibilities in drug development. For example, they offer exceptional flexibility in constructing disease models by incorporating diverse disease and healthy microtissues into this MPS. This allows for the easy construction of complex multimorbidity models, something that is challenging with natural mammal models due to their inability to withstand simultaneous stimulation from multiple pathogens.

It can be observed that this MPS is equivalent to the real rat in terms of “blood” circulation system efficiency, excretion system efficiency, and drug-time curve and efficacy/toxicity of carboplatin. This demonstrates that this MPS could be used as a reliable surrogate in laboratory experiments. The reason behind this capability lies in the design of the system, which includes: (1) co-culture of up to 18 microtissues representing the physiological proportion; (2) customized culture media for 18 microtissues to achieve the superior viability; (3) collagenase pretreatment of freshly-cut microtissues to increase the viability; (4) a precise “blood” circulation system with volumetric flow proportions comparable to in vivo; (5) micro-mixing optimization for efficient removal of harmful wastes from the arterial system. This MPS can be further improved through integration of additional support systems such as digestive, respiratory, and neuro-systems, as well as integration of micro-mixing into each “organ” well. Through this, this MPS can more closely mimic the real rat and reduce reliance on laboratory rat use. This is particularly noteworthy considering that the annual use of rats and mice only in the U.S. reached approximately 111.5 million between 2017 and 2018^80^.

The utilization of the proposed strategy has the potential to substantially reduce dependence on not just rats, but virtually all laboratory animals. For example, we look into cynomolgus monkeys, it becomes evident that a single cynomolgus monkey can produce hundreds of monkey microphysiological systems. This breakthrough holds significant potential to expand the number of experimental drug evaluations while minimizing the number of cynomolgus monkeys needed, thus obtaining more data based on primates and data for the translation from primates to humans. It’s important to acknowledge that while this MPS prototype present promising alternatives, they currently do not cover all types of animal experiments for example, experiments involving animal behavior based on the whole nervous system. Nevertheless, adopting this MPS prototype has the potential to bring about a significant overall reduction in animal experimentation costs.

## Conclusion

In this study, based on the coupling mechanism of animal physiological fluid, an innovative mini mouse multi-organ chip system was constructed. The system is highly integrated with 18 independent organ culture chambers, which can effectively simulate the complex physiological structure and function of the animal body. By constructing such a microphysiological system, we break through the limitations of traditional in vitro research models and achieve a high degree of restoration of animal body and organ interactions and physiological microenvironment in vitro. This highly simulated nature means that the reaction of drugs in the body can be predicted with greater precision.

During the drug screening phase, the system can be utilized to rapidly evaluate the efficacy and safety of a large number of drug candidates. Compared with traditional screening methods, the system can provide rich and accurate drug response data in a short time, greatly shorten the drug screening cycle, and significantly improve the efficiency of research and development, so that more potential drugs can enter the subsequent research and development stage faster. At the same time, by monitoring the metabolic process of drugs in MPS and their effects on different organs and tissues, the mechanism of drug action can be more deeply understood, and the key basis for drug optimization design can be provided.

The benefits of the MPS system are also significant in toxicity studies. At present, the traditional toxicity testing methods often difficult to accurately evaluate the potential toxic effects of drugs on various organs because they cannot fully simulate the synergistic effects of multiple organ systems. The MPS system constructed in this study covers a variety of organ microtissues, just like a miniature animal body, and can comprehensively observe the toxic reactions of drugs to different organs, including the damage of drugs to the liver, kidney, heart, and other important organs. This helps to detect potential toxicity risks at an early stage of drug development, avoid the entry of drugs with severe toxicity into clinical trials, and reduce research and development costs and risks.

In summary, the results of this study not only verify the feasibility and complexity of the MPS method but also provide a new technical platform and research ideas for drug development and toxicity research, which is expected to play an important role in the development process of the future biomedical field.

### Supporting Information

The supporting information is available free of charge via the Internet at http://pubs.acs.org, which include SI.docx incorporating supplementary data and related discussions. In addition, 9 supporting videos can be downloaded from https://figshare.com/articles/media/Videos/27641556.

## Supplementary information

Supplemental Information
