# Supplementary material for: An eighteen-organ microphysiological system coupling a vascular network and excretion system for drug discovery
Source: Microsyst Nanoeng. 2025 May 14;11:89. doi: 10.1038/s41378-025-00933-3 (PMC12078732; doi:10.1038/s41378-025-00933-3)
Supplement: Supplementary file 1 — Supplemental Information [file 41378_2025_933_MOESM1_ESM.docx]

Supporting Information

An “Eighteen-Organ” Microphysiological System Coupling a Vascular Network and Excretion System for Drug Discovery

**1. Supplementary Results and Discussions**

- 1. **“Organ” compartments**


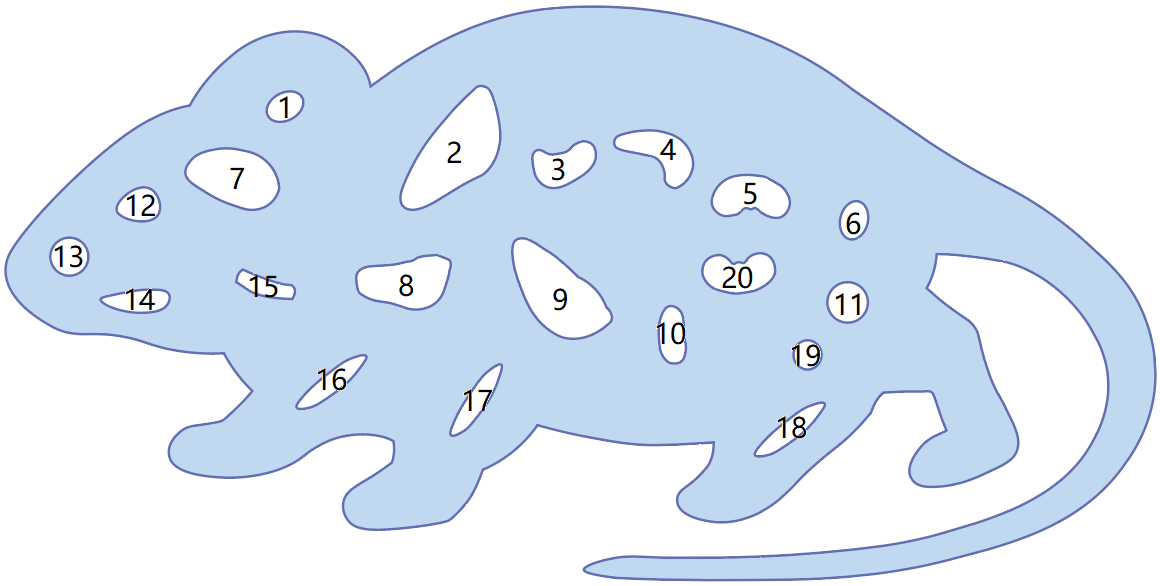


**Figure S1.** **The distribution of the compartments inside the MPS body and location of the 18 kinds of “organs”.** (1) “ear”, (2) “lung”, (3) “stomach”, (4) “pancreas”, (5) “kidney”, (6) “testicle”, (7) “brain”, (8) “heart”, (9) “liver”, (10) “spleen”, (11) tumor, (12) “eye”, (13) “nose”, (14) “tongue”, (15) “trachea”, (16) “muscle”, (17) “adipose”, (18) “marrow”, (19) “skin”, (20) kidney-1

**Table S1. Quantitative description of the compartments in the MPS**

| Microtissue entity | Outline | Brain | 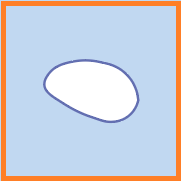 | Eye | 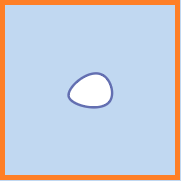 |
| --- | --- | --- | --- | --- | --- |
| Capacity  (µL) |  | 92.0 |  | 25.0 |  |
| Ear | 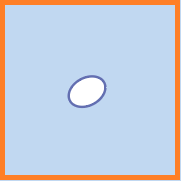 | Nose | 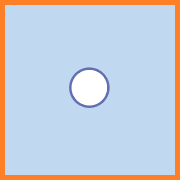 | Tongue | 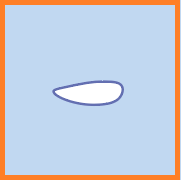 |
| 18.6 |  | 25.1 |  | 26.7 |  |
| Trachea | 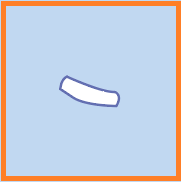 | Heart | 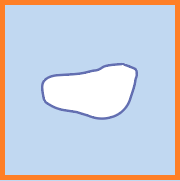 | Lung | 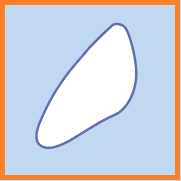 |
| 20.2 |  | 81.9 |  | 149.5 |  |
| Liver | 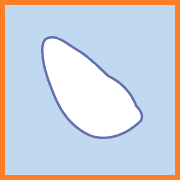 | Stomach | 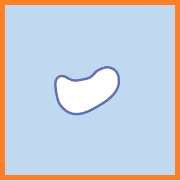 | Pancreas | 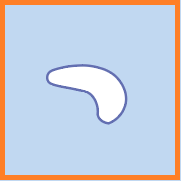 |
| 127.0 |  | 43.5 |  | 51.6 |  |
| Kidney-1 | 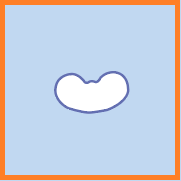 | Kidney | 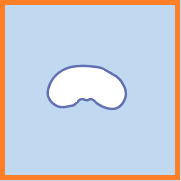 | Spleen | 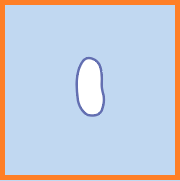 |
| 56.0 |  | 56.0 |  | 28.0 |  |
| Skin | 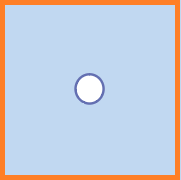 | Adipose | 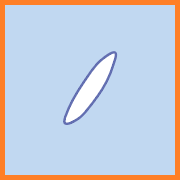 | Marrow | 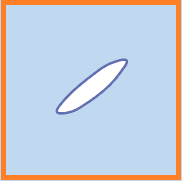 |
| 14.2 |  | 28.8 |  | 28.8 |  |
| Muscle | 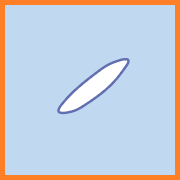 | Testicle | 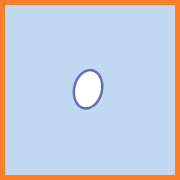 | Tumor | 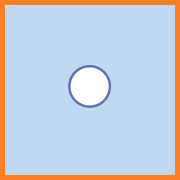 |
| 28.8 |  | 18.6 |  | 28.4 |  |

We cultured primary kidney microtissues in a PMMA well and have observed their attachment to the surface within 24 hours and completely collapsing after 72 hours (Figure S2), indicating that the MPS would fail after 24 hours of culturing. To address this issue, we coated the PMMA surface with a layer of hydrophobic nanoparticles to make it superhydrophobic^1^. The surface modification procedure involved mixing 0.25 g of hydrophobic silica particles (20 nm), 50 ml of n-hexane, and 2.5 ml of chloroform thoroughly, stirring the solution to avoid sedimentation of the nanoparticles, immersing the PMMA substrate in the solution for approximately 15 seconds, and then air-drying it.


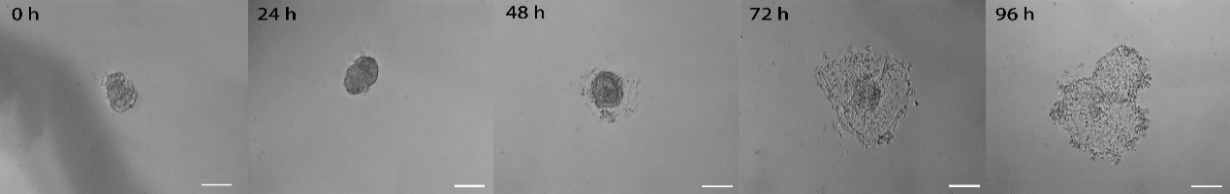


**Figure S2.** **The collapsing process of the primary kidney microtissues on a bare PMMA planar surface.** (scale bar:100µm）

Figure S3 showed the morphology of the superhydrophobic PMMA surface. Video-8 in SI demonstrated the water-proof ability of the superhydrophobic coating. Figure S4 showed that the aqueous solution contracted into a droplet in the superhydrophobic PMMA compartment.

**
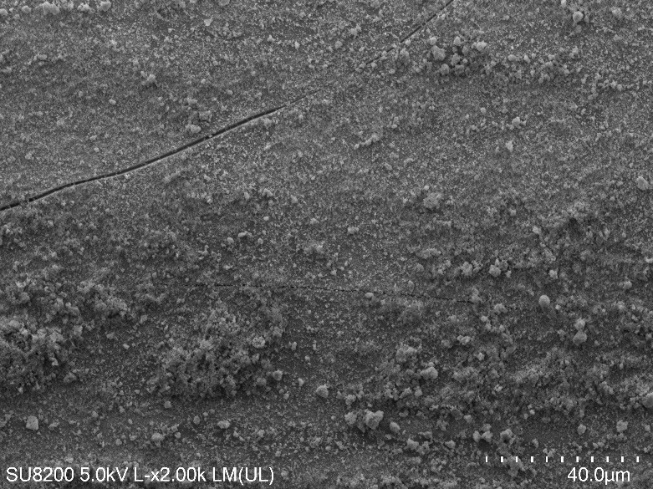
**

**Figure S3.** **The SEM images of the PMMA surface treated with nano silica particles.**

**
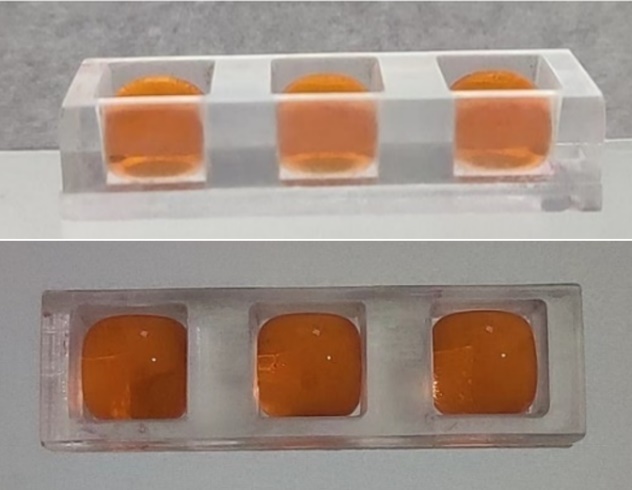
**

**Figure S4.** **The photograph of the aqueous solution in a superhydrophobic PMMA compartment.**


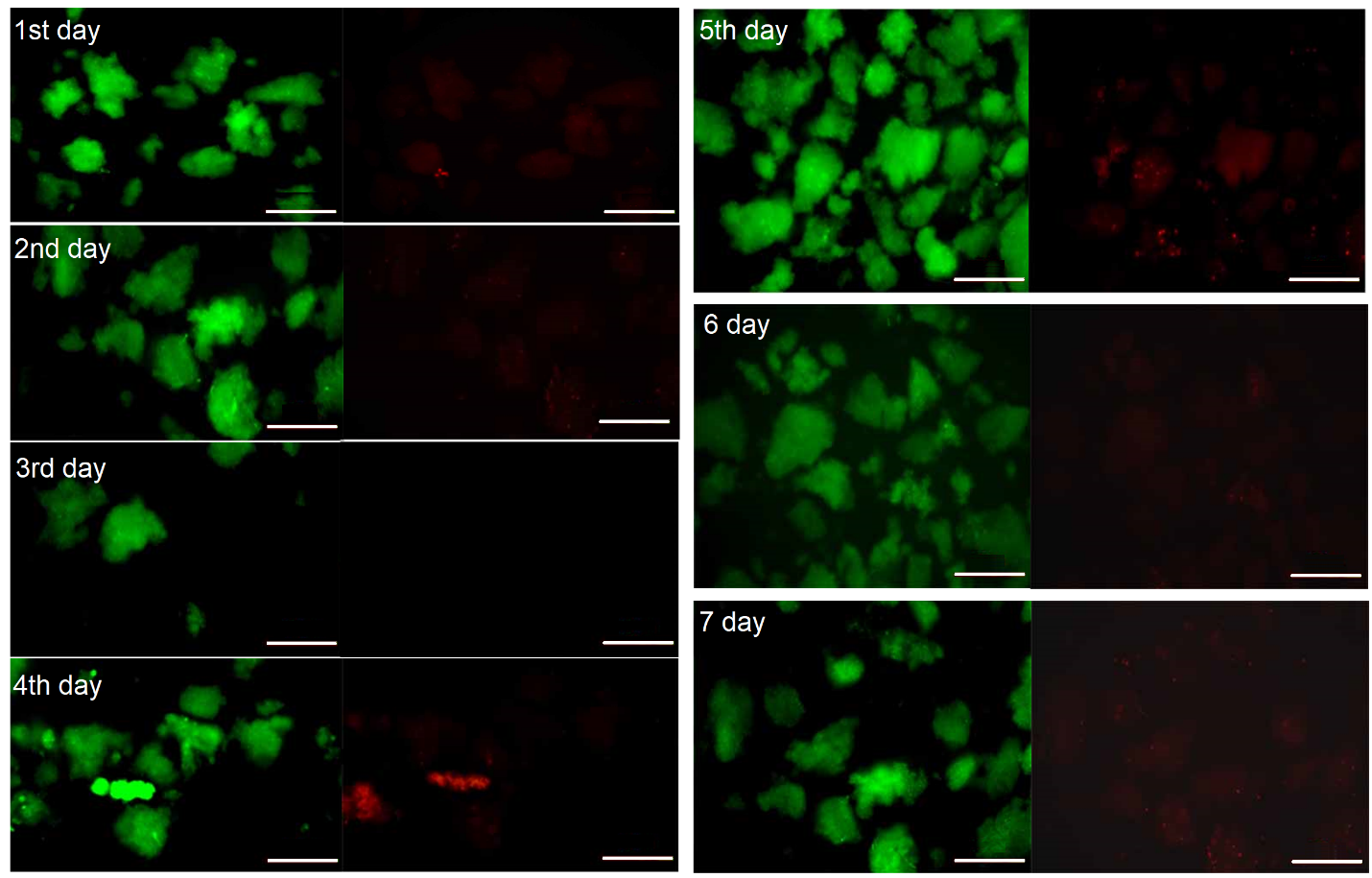


**Figure S5.** **The evolution of the primary kidney microtissues (Live/dead imaging) in a superhydrophobic PMMA well.** (scale bar=100µm)

The superhydrophobic PMMA surface can resist the attachment of primary microtissues for two reasons: (1) the superhydrophobic surface is self-cleaning and can prevent the attachment of large biomolecules like proteins; (2) the aqueous solution contracts on the superhydrophobic surface, minimizing the contact area of the solution and the surface. To demonstrate the working performance of the superhydrophobic surface, we cultured primary kidney microtissues in a superhydrophobic PMMA well and found that they did not attach for at least 7 days; furthermore, the high viability of the kidney microtissues showed the biocompatibility of the superhydrophobic coating (Figure S5).

**1.2. Primary rat microtissues**


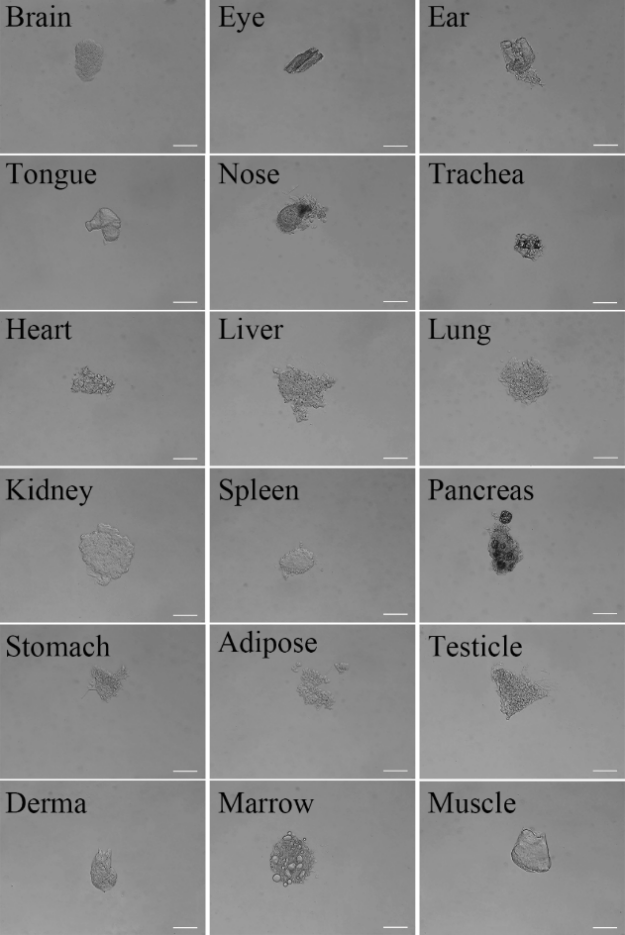


**Figure S6.** **The typical bright-field images of the 18 kinds of the primary microtissues.** (scale bar=100µm)

**Table S2. Differentiated culture medium in MPS**

|  | Culture medium |
| --- | --- |
| Blood | DMEM/F-12 +20% EBM-2(Endothelial Cell Basal Medium-2)+5% FBS(Fetal Bovine Serum)+1% ITS(Insulin-Transferrin-Selenium Ethanolamine additive)+10 ng/mL EGF (Epidermal Growth Factor)+1 μg/mL Hydrocortisone+1% Penicillin-Streptomycin |
| Heart | DMEM/F12+10% FBS +1% penicillin-streptomycin |
| Liver | DMEM High Glucose Medium+10% FBS +1% ITS+1% penicillin-streptomycin |
| Spleen | RPMI 1640 +10% FBS +1% Penicillin-Streptomycin |
| Lung | RPMI 1640+10% FBS+1% Penicillin-Streptomycin |
| Kidney | DMEM/F12 +5% FBS +1% ITS+10 ng/mL EGF+1 μg/mL Hydrocortisone +1% Penicillin-Streptomycin |
| Brain | DMEM/F12 + 5% FBS+1% ITS+1% Penicillin-Streptomycin+1% B-27 Supplement +1%N-2 Supplement |
| Eye | DMEM/F12+5% FBS +1% Penicillin-Streptomycin |
| Tongue | DMEM/F12+5% FBS+1%ITS +1% Penicillin-Streptomycin |
| Nose | DMEM/F12+5% FBS+1% ITS +1% Penicillin-Streptomycin |
| Ear | DMEM/F12+5% FBS+1% Penicillin-Streptomycin |
| Marrow | DMEM/F12+ 10% FBS+1% Penicillin-Streptomycin+1% ITS |
| Skin | DMEM/F12+2% FBS+1% EGF+1% Penicillin-Streptomycin+1% ITS |
| Muscle | DMEM+15% FBS+1% Penicillin-Streptomycin+1% ITS |
| Trachea | DMEM/F12+ 10% FBS+1% Penicillin-Streptomycin |
| Stomach | DMEM/F12 +5% FBS +1% ITS+1% Penicillin-Streptomycin +Hepes+5 ng/mL EGF |
| Fat | DMEM/F12 +20% FBS +1% Penicillin-Streptomycin |
| Testicle | DMEM/F12+5% FBS+1% Penicillin-Streptomycin+1% ITS |
| Pancreas | DMEM/F12 +5% FBS +1% ITS+1% Penicillin-Streptomycin +0.1 mg/mL Trypsin inhibitor +10 ng/mL EGF |

We killed three rats (~124.3g in average) and measured the individual organ mass in average.

**Table S3. The microtissue mass in the chamber**

|  | Brain | Heart | Lung | Liver | Pancreas | Muscle | Kidney | Adipose | Spleen |
| --- | --- | --- | --- | --- | --- | --- | --- | --- | --- |
| Measured organ mass (g) | ~1.49 | ~0.48 | ~0.88 | ~4.48 | ~0.35 | ~5.49 | ~1.12 | ~0.33 | ~0.4 |
| Microtissue mass in the compartment (mg) | 26.6 | 8.6 | 15.7 | 80.0 | 6.3 | 18.0 | 20 | 5.9 | 7.2 |
|  | Eye | Ear | Nose | Tongue | Skin | Marrow | Testicle | Stomach | Trachea |
| Measured organ mass (g) | ~0.18 | ~0.13 | ~0.03 | ~0.03 | ~16.53 | Immeasurable | ~1.02 | ~0.95 | ~0.04 |
| Microtissue mass in the compartment (mg) | 3.2 | 2.3 | 0.6 | 0.6 | 9.00 | 12.8 | 9.0 | 17.0 | 0.7 |

Mechanical cutting (Video-1 in SI) occasionally damaged the microtissues, with the majority of the harm being inflicted upon their surface. The introduction of pretreatment via collagenase displayed the ability to eradicate deceased cells located on the surface, thus enhancing the overall vitality of the microtissues. In one particular experiment, Figure S7 indicated that the viability of liver microtissues increased by roughly 20% as a result of collagenase pretreatment. In this study, we also pretreated brain and kidney microtissues with collagenase.


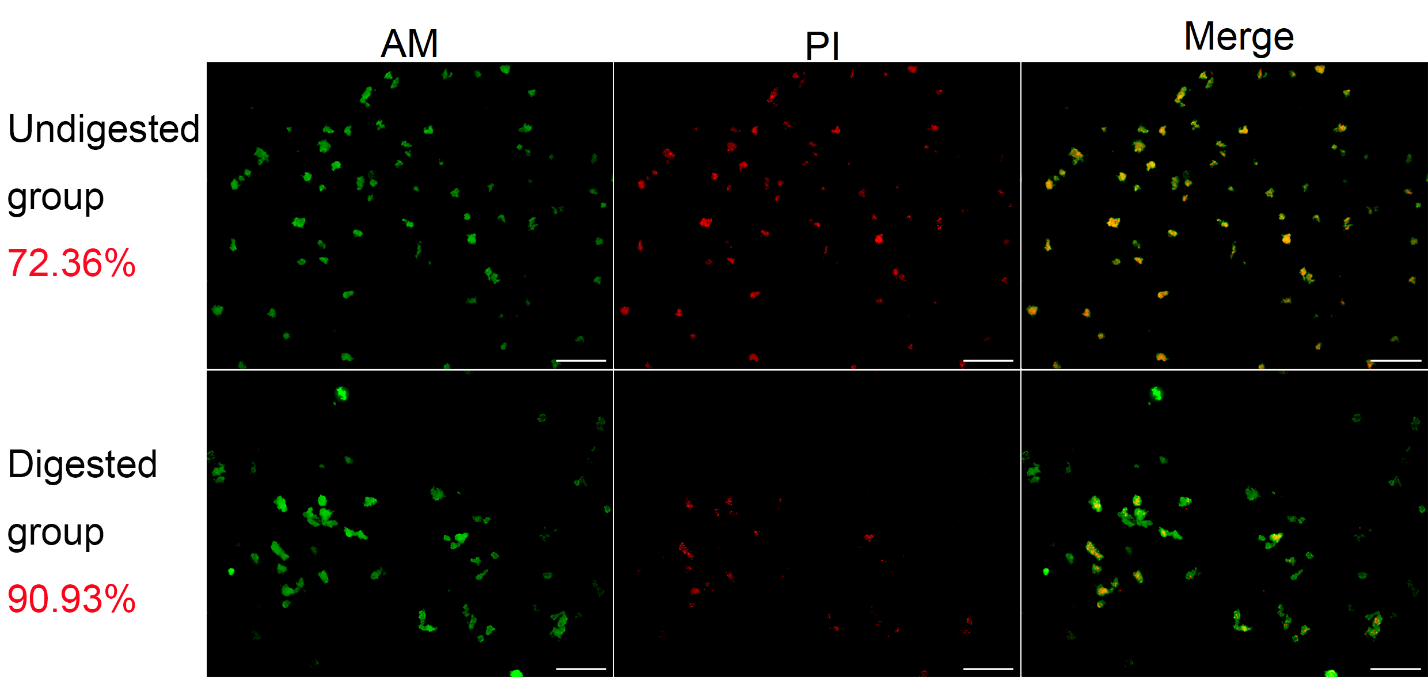


**Figure S7.** **The viability of the liver microtissues before and after the treatment with the collagenase solution.** (Scale bar: 500 µm)


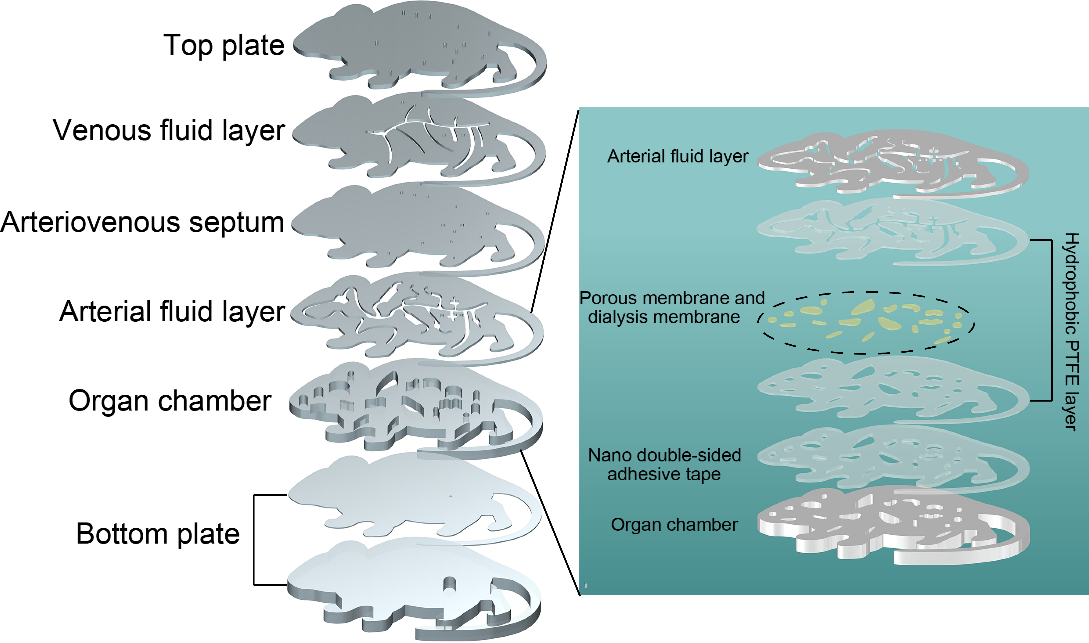


**Figure S8. The exploded view and design details of the MPS**

**1.3. The enhancement of mass transfer inside the kidney-1 compartment by the micro-stirring**

In the MPS, mass transfer between the kidney-1 compartment and artery was primarily diffusion-controlled. To accelerate the mass transfer, we used a DC motor 610 as a micro-stirrer in the compartment. To test the effectiveness of this micro-stirrer, we designed a microdevice (Figure S8), in which the upper chamber had a volume of 200 µL, while the lower chamber had a volume of 452 µL. The lower chamber was filled with fluorescent solution, whereas the upper chamber was filled with water. A porous membrane was sandwiched between the two chambers. We measured the variation of the fluorescent intensity of the water over time. Three different voltages (2.7v, 0.54v, and 0.3v) were applied to the DC motor 610 to achieve high, medium, and low stirring speeds, respectively. Two fluorescent dyes, fluorescent sodium, and 70KD fluorescent dextran, were used to simulate small and large signal molecules, with an initial concentration of 10^-4^ M. It can be observed from Figure S9 that (1) microstirring in the lower chamber enhanced mass transfer between the top chamber and the lower chamber, and (2) mass transfer between the top and the lower chamber was more apparent for small molecules.


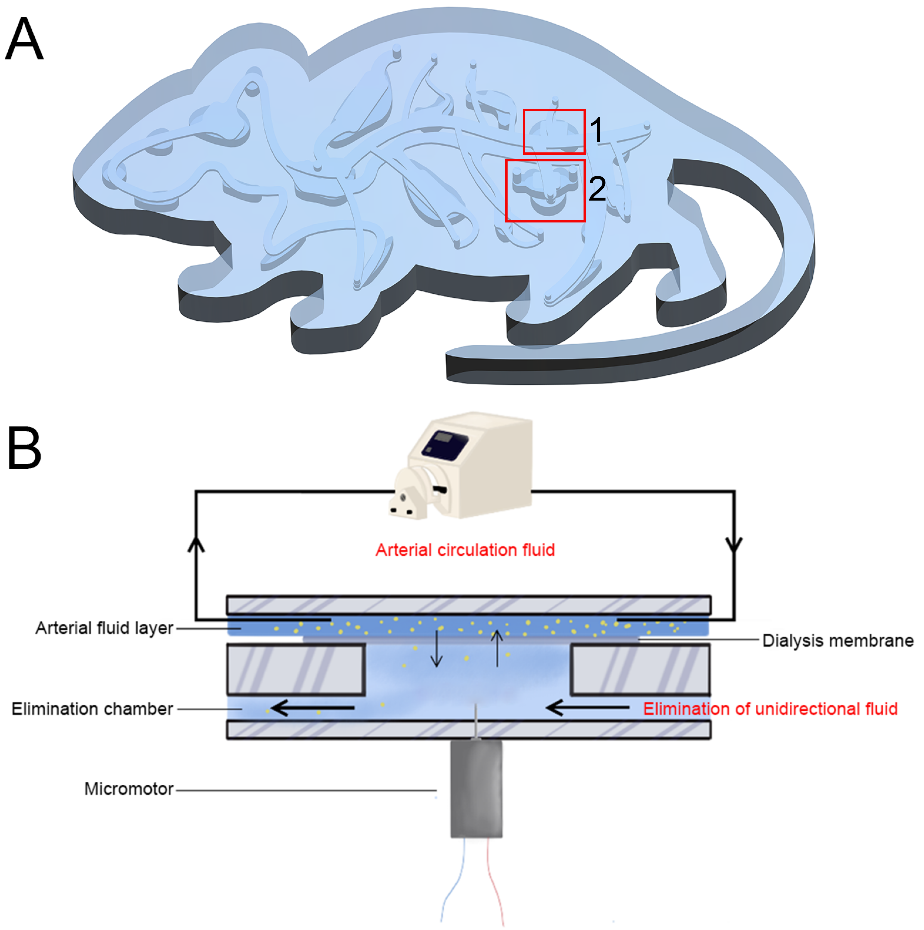


Figure S9 Illustration of kidney elimination and fluid pathway display

**
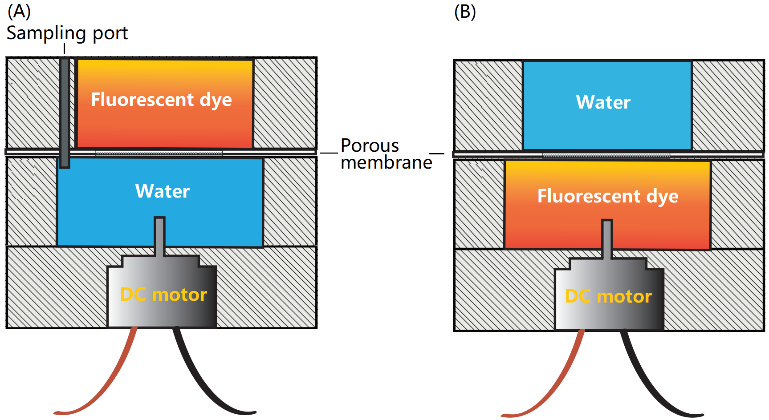
**

**Figure S10.** **Illustration of the microdevice for testifying mass transfer enhancement.**

**
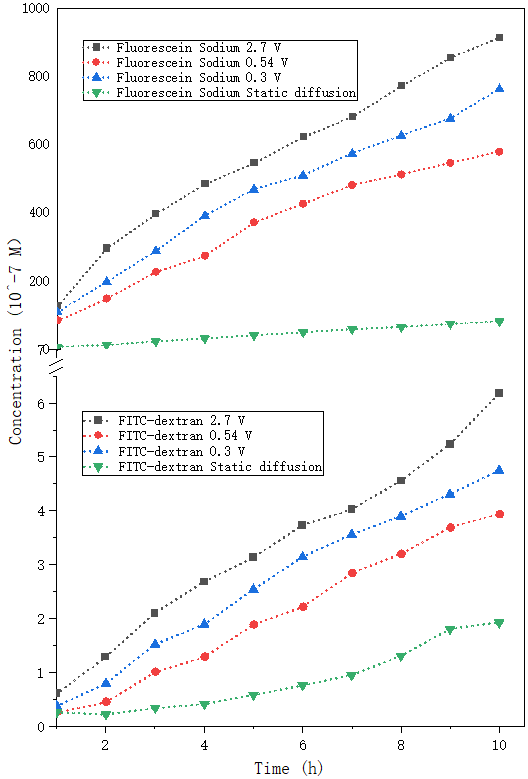
**

**Figure S11.** **Variation of the fluorescent intensity of the water in the microdevice in Figure S8 with time, molecular weight and stirring intensity.**

**1.4. The proportion of the volumetric “blood” flow at each “organ” to the total volumetric “blood” flow in the MPS**


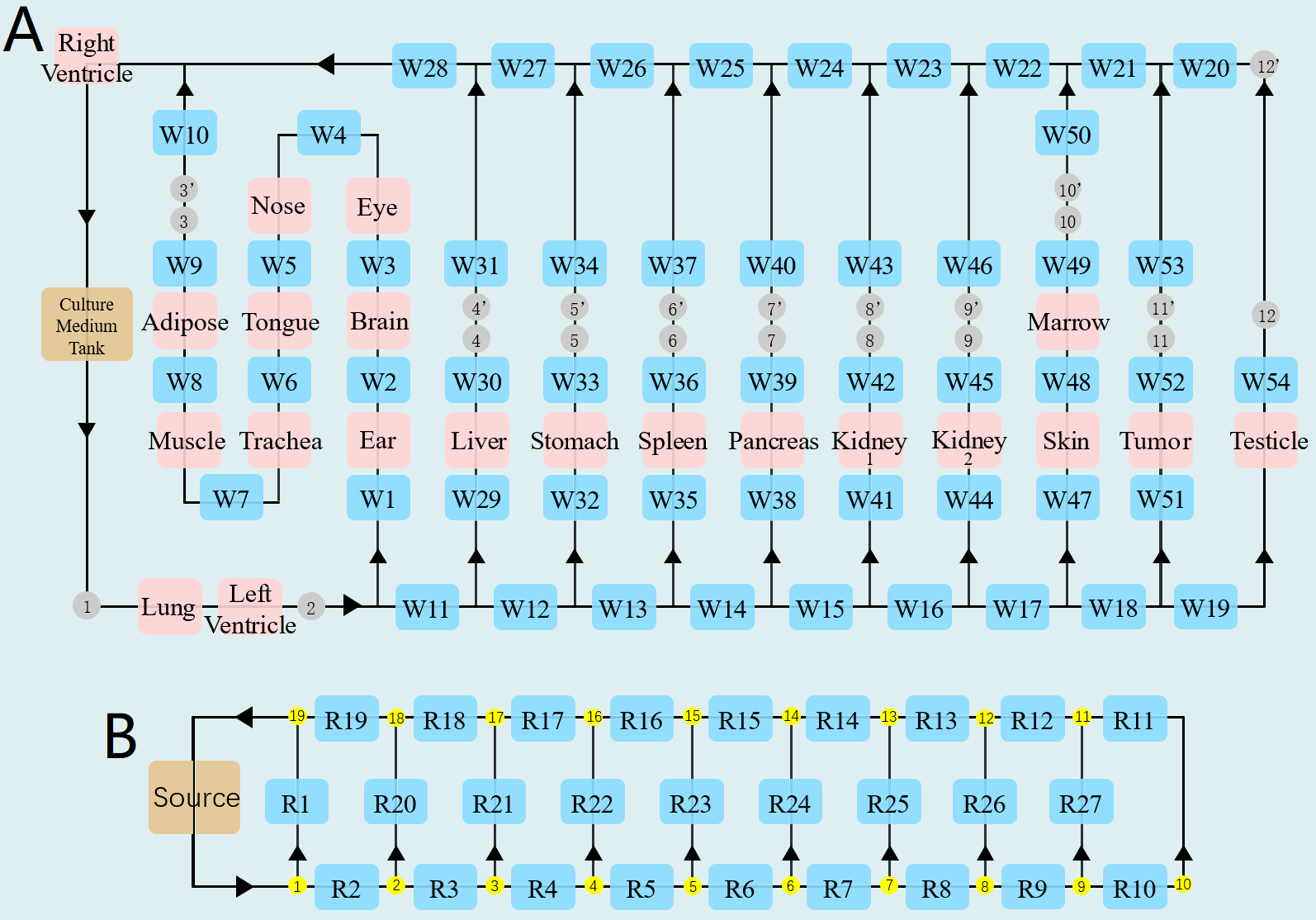


**Figure S12.** **Equivalent fluidic circuit of Figure 2 in main text. All the blocks represent a flow resistance (A) and the simplified form of A frame (B).**

Figure 2 in the main text showed the “blood” circulation system of the MPS, which can be transformed as Figure S10A. Figure S10A can be further simplified as Figure S10 B. And the following equations were available, in which $W_{x}$ was the flow resistance of the microchannel segment in Figure S10A, and $R_{x}$ was the flow resistance of the microchannel segment in Figure S10 B. It can be easily observed that the following equations are available.

$W_{1}+W_{ear}+W_{2}+W_{brain}+W_{3}+W_{eye}+W_{4}+W_{nose}+W_{5}+W_{tongue}+W_{6}+W_{trachea}+W_{7}+W_{muscle}+W_{8}+W_{adipose}+W_{9}+W_{10}=R_{1}$ (01)

$W_{11}=R_{2}$ (02)

$W_{12}=R_{3}$ (03)

$W_{13}=R_{4}$ (04)

$W_{14}=R_{5}$ (05)

$W_{15}=R_{6}$ (06)

$W_{16}=R_{7}$ (07)

$W_{17}=R_{8}$ (08)

$W_{18}=R_{9}$ (09)

$W_{19}+W_{testicle}+W_{54}=R_{10}$ (10)

$W_{20}=R_{11}$ (11)

$W_{21}=R_{12}$ (12)

$W_{22}=R_{13}$ (13)

$W_{23}=R_{14}$ (14)

$W_{24}=R_{15}$ (15)

$W_{25}=R_{16}$ (16)

$W_{26}=R_{17}$ (17)

$W_{27}=R_{18}$ (18)

$W_{28}=R_{19}$ (19)

$W_{29}+W_{liver}+W_{30}+W_{31}=R_{20}$ (20)

$W_{32}+W_{stomach}+W_{33}+W_{34}=R_{21}$ (21)

$W_{35}+W_{spleen}+W_{36}+W_{37}=R_{22}$ (22)

$W_{38}+W_{pancreas}+W_{39}+W_{40}=R_{23}$ (23)

$W_{41}+W_{kidney-1}+W_{42}+W_{43}=R_{24}$ (24)

$W_{44}+W_{kidney}+W_{45}+W_{46}=R_{25}$ (25)

$W_{47}+W_{skin}+W_{48}+W_{marrow}+W_{49}+W_{50}=R_{26}$ (26)

$W_{51}+W_{tumor}+W_{52}+W_{53}=R_{27}$ (27)

$W_{x}=\frac{12\mu L}{1-0.63(H/T)}\cdot\frac{1}{H^{3}T}$ (28)^2^

where $\mu$ was the viscosity, $L$ was the channel length, $H$ was the channel height, and $T$ was the channel width

In Figure S10 B, assuming the potential at the point 1, 2, 3, 4, 5, 6, 7, 8, 9, 10, 11, 12, 13, 14, 15, 16, 17, 18 and 19 were U_1_, U_2_, U_3_, U_4_, U_5_, U_6_, U_7_, U_8_, U_9_, U_10_, U_11_, U_12_, U_13_, U_14_, U_15_, U_16_, U_17_, U_18_ and U_19_, respectively, and the volumetric “blood” flow through R_1_, R_2_, R_3_, R_4_, R_5_, R_6_, R_7_, R_8_, R_9_, R_10_, R_11_, R_12_, R_13_, R_14_, R_15_, R_16_, R_17_, R_18_, R_19_, R_20_, R_21_, R_22_, R_23_, R_24_, R_25_, R_26_ and R_27_ were I_1_, I_2_, I_3_, I_4_, I_5_, I_6_, I_7_, I_8_, I_9_, I_10_, I_11_, I_12_, I_13_, I_14_, I_15_, I_16_, I_17_, I_18_, I_19_, I_20_, I_21_, I_22_, I_23_, I_24_, I_25_, I_26_ and I_27,_ respectively, the following equations governed the volumetric “blood” flows in the MPS,

$U_{19}=0$ (29)

$U_{1}-U_{19}/R_{1}=I_{1}$ (30)

$(U_{1}-U_{2})/R_{2}=I_{2}$ (31)

$(U_{2}-U_{3})/R_{3}=I_{3}$ (32)

$(U_{3}-U_{4})/R_{4}=I_{4}$ (33)

$(U_{4}-U_{5})/R_{5}=I_{5}$ (34)

$(U_{5}-U_{6})/R_{6}=I_{6}$ (35)

$(U_{6}-U_{7})/R_{7}=I_{7}$ (36)

$(U_{7}-U_{8})/R_{8}=I_{8}$ (37)

$(U_{8}-U_{9})/R_{9}=I_{9}$ (38)

$(U_{9}-U_{10})/R_{10}=I_{10}$ (39)

$(U_{10}-U_{11})/R_{11}=I_{11}$ (40)

$(U_{11}-U_{12})/R_{12}=I_{12}$ (41)

$(U_{12}-U_{13})/R_{13}=I_{13}$ (42)

$(U_{13}-U_{14})/R_{14}=I_{14}$ (43)

$(U_{14}-U_{15})/R_{15}=I_{15}$ (44)

$(U_{15}-U_{16})/R_{16}=I_{16}$ (45)

$(U_{16}-U_{17})/R_{17}=I_{17}$ (46)

$(U_{17}-U_{18})/R_{18}=I_{18}$ (47)

${(U}_{18}-U_{19}/R_{19}=I_{19}$ (48)

$(U_{2}-U_{18})/R_{20}=I_{20}$ (49)

$(U_{3}-U_{17})/R_{21}=I_{21}$ (50)

$(U_{4}-U_{16})/R_{22}=I_{22}$ (51)

$(U_{5}-U_{15})/R_{23}=I_{23}$ (52)

$(U_{6}-U_{14})/R_{24}=I_{24}$ (53)

$(U_{7}-U_{13})/R_{25}=I_{25}$ (54)

$(U_{8}-U_{12})/R_{26}=I_{26}$ (55)

$(U_{9}-U_{11})/R_{27}=I_{27}$ (56)

$I_{3}+I_{20}=I_{2}$ (57)

$I_{4}+I_{21}=I_{3}$ (58)

$I_{5}+I_{22}=I_{4}$ (59)

$I_{6}+I_{23}=I_{5}$ (60)

$I_{7}+I_{24}=I_{6}$ (61)

$I_{8}+I_{25}=I_{7}$ (62)

$I_{9}+I_{26}=I_{8}$ (63)

$I_{10}+I_{27}=I_{9}$ (64)

$I_{11}=I_{10}$ (65)

$I_{11}+I_{27}=I_{12}$ (66)

$I_{12}+I_{26}=I_{13}$ (67)

$I_{13}+I_{25}=I_{14}$ (68)

$I_{14}+I_{24}=I_{15}$ (69)

$I_{15}+I_{23}=I_{16}$ (70)

$I_{16}+I_{22}=I_{17}$ (71)

$I_{17}+I_{21}=I_{18}$ (72)

$I_{18}+I_{20}=I_{19}$ (73)

And the final proportion can be expressed by the following equation

$P=\frac{I_{organ}}{\left( I_{1}+I_{19} \right)}$(74) In our MPS, we found when the dimensions of the microchannel followed the values below, the organ blood flow distribution would agree with the *in vivo* condition.

$H_{1}=1.0,T_{1}=1.1,L_{1}=17.2$ (75)

$H_{2}=1.0,T_{2}=1.1,L_{2}=4.6$ (76)

$H_{3}=1.0,T_{3}=1.1,L_{3}=6.3$ (77)

$H_{4}=1.0,T_{4}=1.1,L_{4}=5.5$ (78)

$H_{5}=1.0,T_{5}=1.1,L_{5}=5.8$ (79)

$H_{6}=1.0,T_{6}=1.1,L_{6}=13.5$ (80)

$H_{7}=1.0,T_{7}=1.1,L_{7}=14.4$ (81)

$H_{8}=1.0,T_{8}=1.1,L_{8}=14.0$ (82)

$H_{9}=1.0,T_{9}=1.1,L_{9}=3.1$ (83)

$H_{10}=1.0,T_{10}=1.2,L_{10}=20.5$ (84)

$H_{11}=1.0,T_{11}=2.0,L_{11}=8.8$ (85)

$H_{12}=1.0,T_{12}=2.0,L_{12}=3.1$ (86)

$H_{13}=1.0,T_{13}=2.0,L_{13}=10.3$ (87)

$H_{14}=1.0,T_{14}=2.0,L_{14}=6.8$ (88)

$H_{15}=1.0,T_{15}=2.0,L_{15}=6.3$ (89)

$H_{16}=1.0,T_{16}=2.0,L_{16}=1.4$ (90)

$H_{17}=1.0,T_{17}=2.0,L_{17}=4.2$ (91)

$H_{18}=1.0,T_{18}=2.0,L_{18}=3.2$ (92)

$H_{19}=1.0,T_{19}=2.0,L_{19}=7.5$ (93)

$H_{20}=1.0,T_{20}=2.0,L_{20}=5.9$ (94)

$H_{21}=1.0,T_{21}=2.0,L_{21}=3.5$ (95)

$H_{22}=1.0,T_{22}=2.0,L_{22}=6.1$ (96)

$H_{23}=1.0,T_{23}=2.0,L_{23}=2.1$ (97)

$H_{24}=1.0,T_{24}=2.0,L_{24}=9.2$ (98)

$H_{25}=1.0,T_{25}=2.0,L_{25}=4.6$ (99)

$H_{26}=1.0,T_{26}=2.0,L_{26}=8.7$ (100)

$H_{27}=1.0,T_{27}=2.0,L_{27}=6.6$ (101)

$H_{28}=1.0,T_{28}=2.0,L_{28}=5.1$ (102)

$H_{29}=1.0,T_{29}=1.0,L_{29}=4.5$ (103)

$H_{30}=1.0,T_{30}=1.0,L_{30}=10.7$ (104)

$H_{31}=1.0,T_{31}=1.0,L_{31}=20.3$ (105)

$H_{32}=0.5,T_{32}=0.6,L_{32}=7.3$ (106)

$H_{33}=0.5,T_{33}=0.6,L_{33}=10.4$ (107)

$H_{34}=1.0,T_{34}=1.0,L_{34}=12.6$ (108)

$H_{35}=0.5,T_{35}=0.8,L_{35}=12.3$ (109)

$H_{36}=0.5,T_{36}=0.8,L_{36}=8.0$ (110)

$H_{37}=1.0,T_{37}=1.0,L_{37}=23.6$ (111)

$H_{38}=0.5,T_{38}=0.6,L_{38}=9.0$ (112)

$H_{39}=0.5,T_{39}=0.6,L_{39}=7.0$ (113)

$H_{40}=1.0,T_{40}=1.0,L_{40}=11.8$ (114)

$H_{41}=1.0,T_{41}=1.5,L_{41}=2.4$ (115)

$H_{42}=1.0,T_{42}=1.5,L_{42}=2.2$ (116)

$H_{43}=1.0,T_{43}=2.0,L_{43}=9.3$ (117)

$H_{44}=0.5,T_{44}=0.8,L_{44}=3.7$ (118)

$H_{45}=0.5,T_{45}=0.8,L_{45}=2.4$ (119)

$H_{46}=1.0,T_{46}=1.0,L_{46}=6.8$ (120)

$H_{47}=1.0,T_{47}=2.0,L_{47}=9.9$ (121)

$H_{48}=1.0,T_{48}=2.0,L_{48}=5.8$ (122)

$H_{49}=1.0,T_{49}=2.0,L_{49}=1.9$ (123)

$H_{50}=1.0,T_{50}=2.0,L_{50}=30.5$ (124)

$H_{51}=0.5,T_{51}=1.0,L_{51}=6.4$ (125)

$H_{52}=0.5,T_{52}=1.0,L_{52}=3.3$ (126)

$H_{53}=1.0,T_{53}=1.0,L_{53}=14.1$ (127)

$H_{54}=1.0,T_{54}=2.0,L_{54}=3.3$ (128)

$H_{stomach}=0.5,T_{stomach}=2.8,L_{stomach}=3.5$ (129)

$H_{liver}=1.0,T_{liver}=4.0,L_{liver}=9.0$ (130)

$H_{brain}=1.0,T_{brain}=6.7,L_{brain}=4.0$ (131)

$H_{pancreas}=0.5,T_{pancreas}=4.1,L_{pancreas}=5.2$ (132)

$H_{spleen}=0.5,T_{spleen}=2.7,L_{spleen}=3.6$ (133)

$H_{kidney-1}=1.0,T_{kidney-1}=4.3,L_{kidney-1}=2.6$ (134)

$H_{kidney}=0.5,T_{kidney-2}=4.3,L_{kidney-2}=2.6$ (135)

$H_{tumor}=0.5,T_{tumor}=3.8,L_{tumor}=3.8$ (136)

$H_{ear}=1.0,T_{ear}=1.1,L_{ear}=4.0$ (137)

$H_{tongue}=1.0,T_{tongue}=1.1,L_{tongue}=4.0$ (138)

$H_{nose}=1.0,T_{nose}=1.1,L_{nose}=3.9$ (139)

$H_{eye}=1.0,T_{eye}=1.1,L_{eye}=3.6$ (140)

$H_{muscle}=1.0,T_{muscle}=1.1,L_{muscle}=7.6$ (141)

$H_{adipose}=1.0,T_{adipose}=1.1,L_{adipose}=6.0$ (142)

$H_{trachea}=1.0,T_{trachea}=1.1,L_{trachea}=4.0$ (143)

$H_{testicle}=1.0,T_{testicle}=2.0,L_{testicle}=4.2$ (144)

$H_{marrow}=1.0,T_{marrow}=2.0,L_{marrow}=5.9$ (145)

$H_{skin}=1.0,T_{skin}=2.0,L_{skin}=2.9$ (146)

(146)


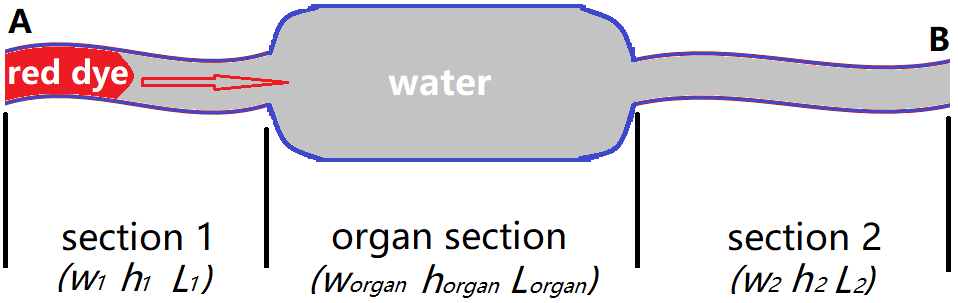


**Figure S13.** **Experimental design for measuring the volumetric “blood” flow.**

To accurately measure the volumetric blood flow in each organ within the MPS, a two-step process was employed. Firstly, pure water was introduced into the microchannel network. Subsequently, a red dye solution was injected from the "lung" port with a constant volumetric flow rate ($I_{total}$). This solution flowed through each "organ" in the microchannel network, and the entire process was recorded in Video-6 in SI. The obtained video was analyzed to determine the volumetric “blood” flow rate at each organ as well as the proportion of “blood” flow at each organ to the total volumetric “blood” flow in the MPS.

As demonstrated in Figure S11, we recorded the arriving time of the red dye solution at the point A and point B, named as $t_{1}$ and $t_{2}$. The volumetric “blood” flow at this “organ”, $I_{organ}$, can be calculated by the following equation

$I_{organ}=\frac{w_{1}\cdot h_{1}\cdot L_{1}+w_{organ}\cdot h_{organ}\cdot L_{organ}+w_{2}\cdot h_{2}\cdot L_{2}}{t_{2}-t_{1}}$ (147)

where $L$ was the channel length, $h$ was the channel height, and $w$ was the channel width. Thus, the volumetric “blood” flow ratio at this “organ”, $P$, can be calculated by the following equation

$P=\frac{I_{organ}}{I_{total}}$ (148)

**1.5. Theoretical calculation of the correlation of drug toxicity and distribution**

We used the ratio of the death rate of the tissue to the concentration of the accumulated drug in the microtissue, $Q$, to characterize the relation of the drug toxicity and distribution at a time point,

$Q=\frac{Tox}{\int_{0}^{t} Dis\cdot dt}$ (149)

Where $Tox$ is the death rate of the tissue, and $Dis$ is the instant drug concentration in the microtissue

$Tox=\frac{D}{N}$ (150)

where $N$ the total number of the cells in an organ, and $D$ is the number of dead cells, defined as

$D=\int_{0}^{t} {V_{c}d}_{t}$ (151)

where $V_{c}$ is the cell death rate, and $t$ is the time

$Dis=\frac{M_{d}}{M_{t}}$ (152)

where $M_{d}$ is the mass of drug in the organ, and $M_{t}$ is the mass of the cells in the organ

$M_{d}=∯_{0}^{S} \int_{0}^{t} \tau(t,S)dt\cdot dS$ (153)

Where $\tau(t,S)$ is the drug flux through the “blood” vessel wall, and $S$ is the “blood” vessel wall in the organ.

$M_{t}$ can be described as the following equation,

$M_{t}=aN$ (154)

where$a$ is a constant, which can be described as,

$a=\frac{w}{1-q}$ (155)

where $q$ is the proportion of the extracellular matrix in the organ, and $w$ is the mass of a single cell,

$w=\rho V$ (156)

Where $\rho$ is the density, and $V$ is the volume of a single cell

Combining the equation of (149)-(156),

$Q=\frac{\rho V\int_{0}^{t} V_{c}d_{t}}{(1-q)\int_{0}^{t} \left[ ∯_{0}^{S} \int_{0}^{t} \tau\left( t,S \right)dt.dS \right]dt}$ (157)


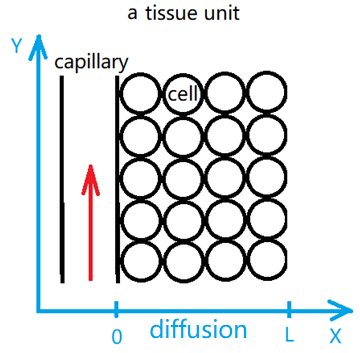


**Figure S14.** **An illustration of a tissue unit.**

In a tissue unit (Figure S14), the transportation of the drug from the blood to the tissue can be regarded as a diffusion-controlled process, in which

$\frac{\partial J(t,x,S)}{\partial x}=-\frac{\partial C(t,x,S)}{\partial t}$ (158)

where $J(t,x,S)$ is the drug flux in the tissue unit, and $C(t,x,S)$ is the concentration of the drug in the tissue unit, and

$J(t,x,S)=\int-\frac{\partial C(x,t,S)}{\partial t}dx$ (159)

$\tau(t,S)=J(t,0,S)$ (160)

According to the equation (157), (159), (160), we obtained

$Q=\frac{\rho V\int_{0}^{t} V_{c}d_{t}}{(1-q)\int_{0}^{t} \left\{ ∯_{0}^{S} \int_{0}^{t} \left[ \int-\frac{\partial C(x,t,S)}{\partial t}dx \right]dt\cdot dS \right\}dt} [x=0]$ (161)

in which $C(x,t,S)$ can be calculated by,

$\frac{\partial C(x,t,S)}{\partial t}=D\frac{\partial^{2}C(x,t,S)}{\partial x^{2}}$ (162)

$D=D_{Dif}+D_{Act}$ (163)

where $D$ is the apparent diffusion coefficient including passive diffusion term$,D_{Dif}$ and active transportation term, $D_{Act}$.


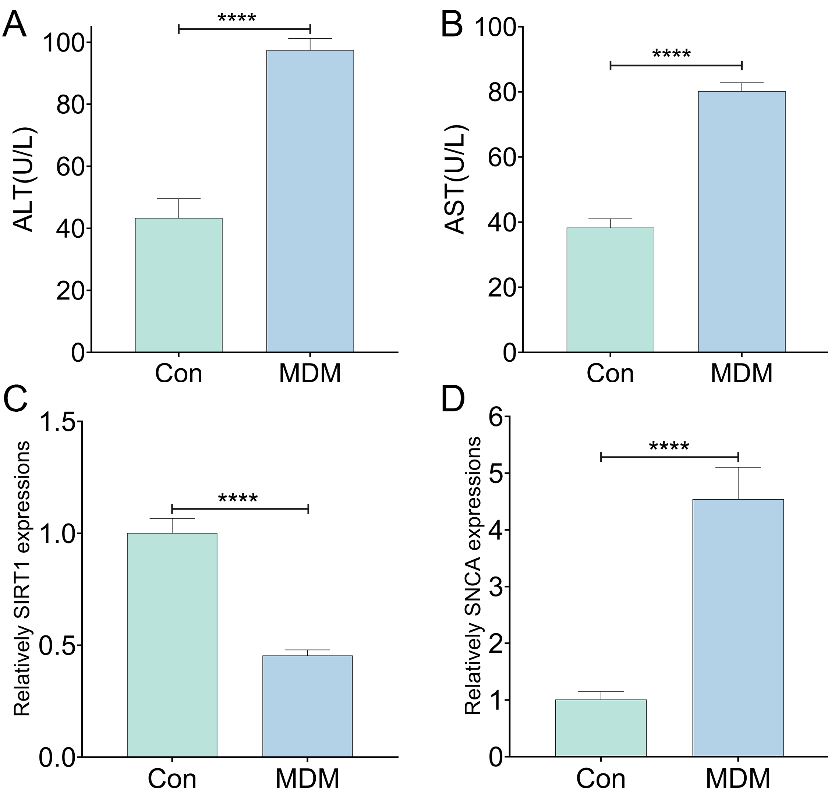


**Figure S15. Validation of senile disease model based on MPS in vitro**

In this study, we combined brain microtissues from elderly rats with Parkinson's disease, liver microtissues from elderly rats with carbon tetrachloride-induced liver injury, and microtissues from various organs such as the heart, lungs, kidneys, muscles, pancreas, adipose tissue, and spleen from normal elderly rats. This allowed us to successfully create an old rat model presenting both Parkinson's disease and liver injury.

To verify the feasibility of the senile disease model built based on MPS, the contents of ALT and AST, as well as α-synucleinand Sirtuin 1 in the microphysiological system were measured after the MPS had been running for 24 hours. Experimental data showed that, compared with the normal model group (Con), ALT and AST levels in the in vitro senile multi-disease model built based on the MPS showed an extremely significant increase trend (P < 0.0001). At the same time, the expression of SIRT1 was significantly down-regulated. As a type III protein deacetylase, SIRT1 has been recognized as a new anti-aging protein, which plays a key role in cell aging and inflammation regulation [4]. Studies have shown that the expression level and enzyme activity of SIRT1 gradually decrease with age [5]. In addition, SNCA expression increased significantly in this model, which is highly consistent with the gene expression pattern characteristic of Parkinson's disease. The SNCA gene is the first autosomal dominant gene identified to be associated with Parkinson's disease, and its overexpression has been confirmed to be an important mechanism of Parkinson's disease [6]. In summary, the results of this experiment strongly prove that we have successfully simulated in vitro animal models with characteristics of liver injury, aging, and Parkinson's disease on the MPS chip platform.

**1.6. The immune cells in the MPS**

The primary rat neutrophils were extracted from the rat blood (Figure S16A). They showed strong chemotaxis in a microfluidic device (Figure S16 B, C, D, E). We added the neutrophils into the culture medium tank, and theses immune cells were then circulating in the “blood”. If using the commercial peristaltic pump to drive the “blood” circulation in the MPS, the neutrophils will experience multiple squeezes each time passing through the peristaltic pump (Figure S17 A), and thus lose some phenotypes and functions. To address this issue, we used microfluidic pneumatic peristaltic pump^3^ (Figure S17 B). We made a such pump driver in lab (Figure S18) to drive the “blood” circulation (Video-9 in SI), in which the squeeze effect can be minimized (Figure S19).


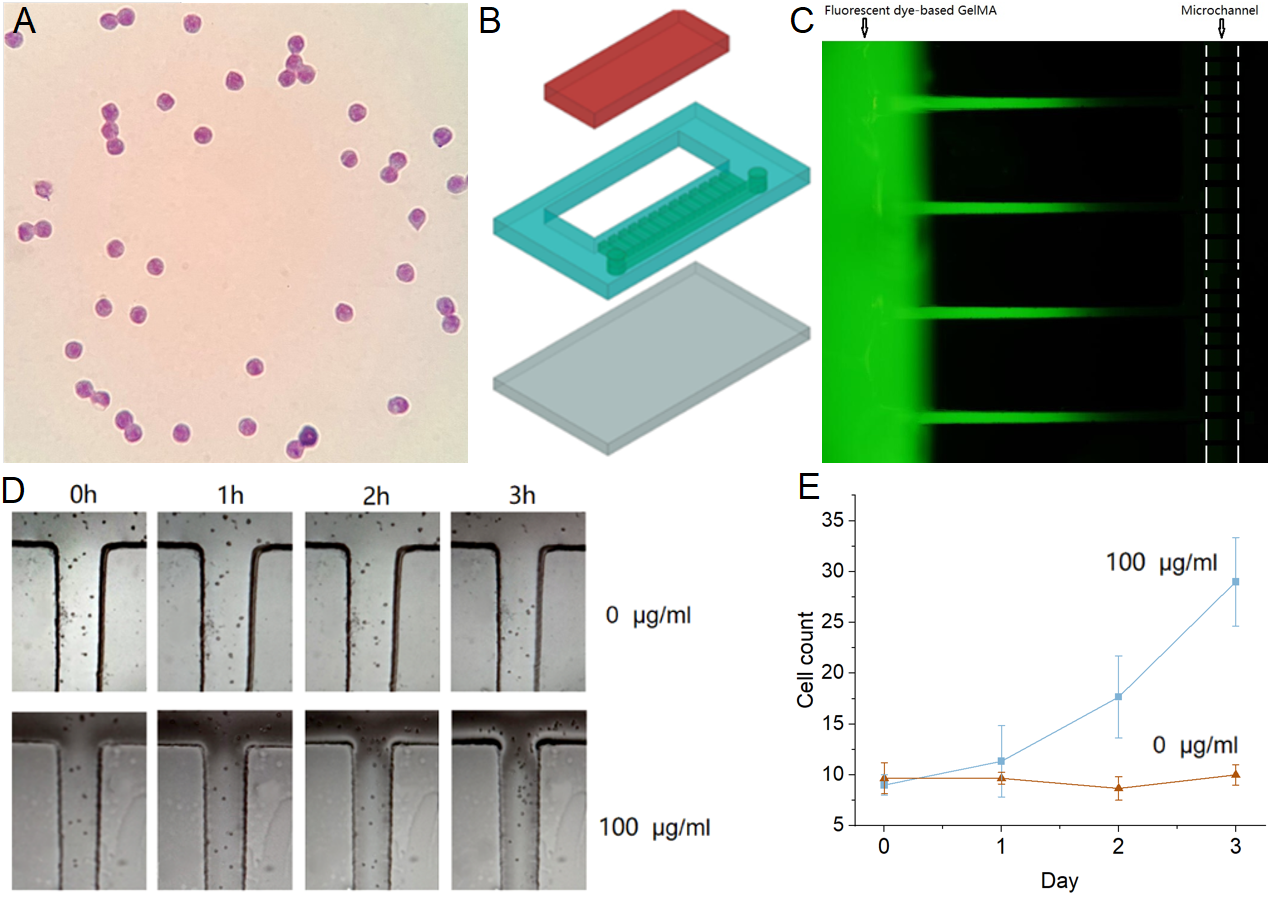


**Figure S16. The characterization of the primary neutrophils extracted from a rat.** (A) Wright-Giemsa staining; (B) Exploded view of the chemotaxis microfluidic device. The brown block represents a GelMA hydrogel monolith containing chemokines (TGF-β) or fluorescent dyes. The middle green layer contains a PDMS microchannel array perpendicular to an adjacent microchannel, housing the primary neutrophils. A central chamber in the middle layer accommodates the GelMA hydrogel monolith. The chemokines or fluorescent dye within the GelMA hydrogel monolith diffuse into the adjacent microchannel through the microchannel array, thereby inducing the chemotaxis of the primary neutrophils. The bottom plate consists of a glass slide. This design effectively mitigates the impact of normal hydrostatic flow in the microchannel array on chemotaxis; (C) Concentration gradient of the fluorescent dye within the microchannel array; (D) Bright field images capturing the neutrophils within the microchannel array at different time points; (E) Statistical analysis of the images shown in frame D.


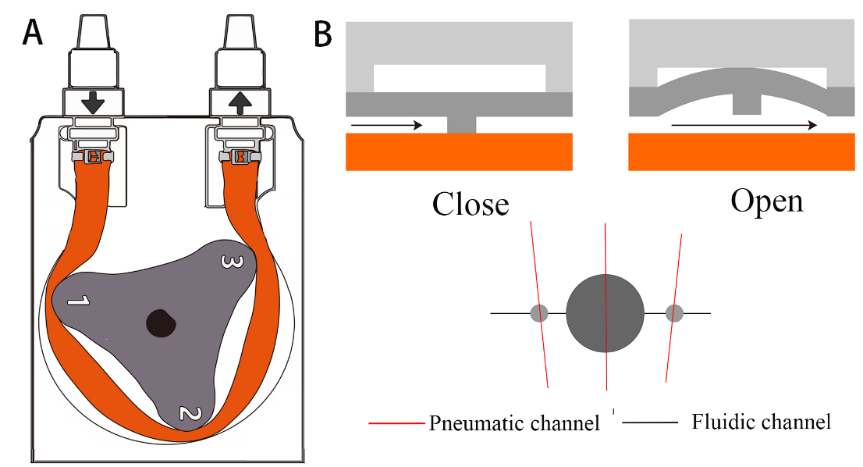


**Figure S17. Comparison of two pumping modes.** (A) Illustration of the traditional peristaltic pump in which the primary immune cells would be squeezed at the point 1, 2, 3; (B) Illustration of the microfluidic pneumatic peristaltic pump in which the squeeze effect was free.


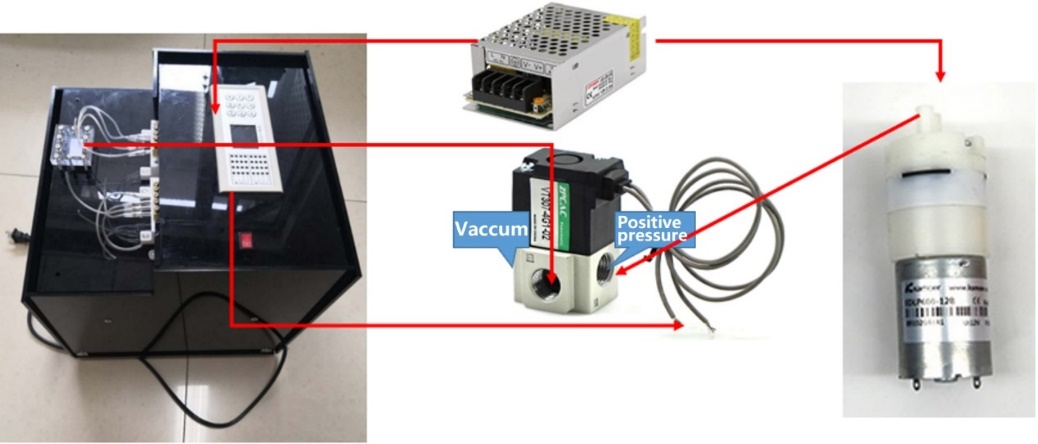


**Figure S18.** Photograph of the home-made microfluidic pneumatic pump driver.


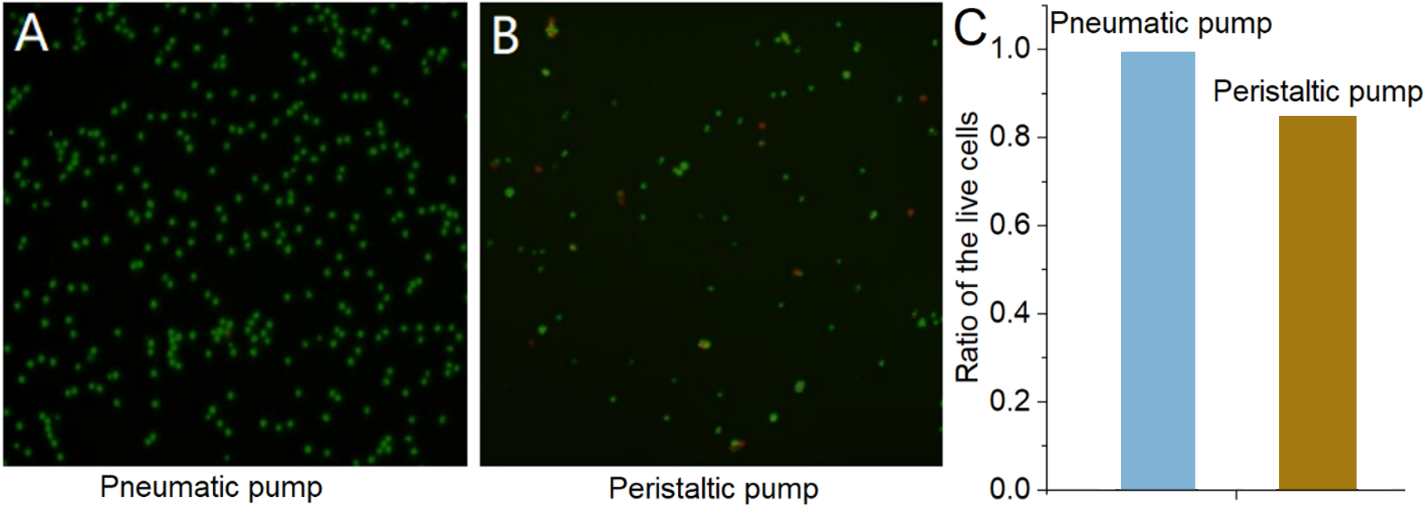


**Figure S19. Effect of the two pumping modes on the viability of the primary neutrophils.** (A) The typical Live/dead fluorescence image of primary neutrophils after one fluidic cycle using microfluidic pneumatic pump; (B) The typical Live/dead fluorescence image of primary neutrophils after one fluidic cycle using normal peristaltic pump; (C) The statistical analysis of the frame A and B.

2. REFERENCE

[1] Chunhui, Z., Moyuan, C., Hongyu, M., Cunlong, Y., Kan, L., Cunming, Y., Lei, J., Morphology-Control Strategy of the Superhydrophobic Poly(Methyl Methacrylate) Surface for Efficient Bubble Adhesion and Wastewater Remediation, Advanced functional materials, 2017,27(43), 8

[2] Conlisk, A. Introduction to Microfluidics. By Patrick Tabeling. Oxford University Press, 2005. 312 pp. ISBN 019 856864 9. Journal of Fluid Mechanics, 2007,570, 503-505.

[3] Grover, W. H., Skelley, A. M., Liu, C. N., Lagally, E. T. and Mathies, R. A. Monolithic Membrane Valves and Diaphragm Pumps for Practical Large-Scale Integration into Microfluidic Devices. Sens. Actuators, B, 89, 315–323 (2003)

[4] Sousa, C., & Mendes, A. F. Monoterpenes as Sirtuin‐1 Activators: Therapeutic Potential in Aging and Related Diseases. In Biomolecules,2022, 12(7).

[5] Vázquez-Vélez, G. E., Gonzales, K. A., Revelli, J. P., Adamski, C. J., Naini, F. A., Bajić, A., Craigen, E., Richman, R., Heman-Ackah, S. M., Wood, M. J. A., Rousseaux, M. W. C., & Zoghbi, H. Y. Doublecortin-like Kinase 1 Regulates α-Synuclein Levels and Toxicity. Journal of Neuroscience, 2020, 40(2), 459–477.

[6] Yao, H., & Rahman, I. Perspectives on translational and therapeutic aspects of SIRT1 in inflammaging and senescence. In Biochemical Pharmacology, 2012, 84(10), 1332–1339.
